# Characterization of *Agrobacterium tumefaciens* PPKs reveals the formation of oligophosphorylated products up to nucleoside nona-phosphates

**DOI:** 10.1007/s00253-020-10891-7

**Published:** 2020-10-06

**Authors:** Celina Frank, Attila Teleki, Dieter Jendrossek

**Affiliations:** 1 Institute of Microbiology, University of Stuttgart, Allmandring 31, 70569 Stuttgart, Germany; 2 Institute of Biochemical Engineering, University of Stuttgart, Stuttgart, Germany

**Keywords:** Polyphosphate, Polyphosphate kinase, Agrobacterium tumefaciens, Nucleotides

## Abstract

**Abstract:**

*Agrobacterium tumefaciens* synthesizes polyphosphate (polyP) in the form of one or two polyP granules per cell during growth. The *A. tumefaciens* genome codes for two polyphosphate kinase genes, *ppk1*_*AT*_ and *ppk2*_*AT*_, of which only *ppk1*_*AT*_ is essential for polyP granule formation in vivo. Biochemical characterization of the purified PPK1_AT_ and PPK2_AT_ proteins revealed a higher substrate specificity of PPK1_AT_ (in particular for adenine nucleotides) than for PPK2_AT_. In contrast, PPK2_AT_ accepted all nucleotides at comparable rates. Most interestingly, PPK2_AT_ catalyzed also the formation of tetra-, penta-, hexa-, hepta-, and octa-phosphorylated nucleosides from guanine, cytosine, desoxy-thymidine, and uridine nucleotides and even nona-phosphorylated adenosine. Our data—in combination with in vivo results—suggest that PPK1_AT_ is important for the formation of polyP whereas PPK2_AT_ has the function to replenish nucleoside triphosphate pools during times of enhanced demand. The potential physiological function(s) of the detected oligophosphorylated nucleotides await clarification.

**Key points:**

•*PPK1*_*AT*_ *and PPK2*_*AT*_ *have different substrate specificities,*

*•PPK2*_*AT*_ *is a subgroup 1 member of PPK2s,*

*•PPK2*_*AT*_ *catalyzes the formation of polyphosphorylated nucleosides*

**Electronic supplementary material:**

The online version of this article (10.1007/s00253-020-10891-7) contains supplementary material, which is available to authorized users.

## Introduction

Polyphosphate (polyP) is an inorganic polymer in which phosphate residues are linked by energy-rich phosphoanhydride bonds. It can be formed either abiotically (vulcanism) or biologically by the action of polyP kinases (PPKs in prokaryotes) or other enzymes (in eukaryotes). PolyP is ubiquitously distributed in species of all domains of life (Kornberg et al. 1999; Rao et al. 2009; Kulakovskaya et al. 2014). In most prokaryotes, polyP is present in form of granule-like inclusions (polyP granules or voluntin granules) with diameters mostly in the range of 50 to 200 nm. In yeasts, polyP can be accumulated up to ≈ one quarter of the cellular weight in vacuole-like compartments (Christ et al. 2020a). PolyP represents a reservoir of phosphorous and apparently is also involved in various forms of stress resistance such as tolerance against heavy metals, elevated temperature, or reactive oxygen species and can fulfill functions in virulence, motility, biofilm formation, and cell cycle control (Rashid and Kornberg 2000; Rashid et al. 2000; Nikel et al. 2013; Chuang et al. 2013; Gray and Jakob 2015; Racki et al. 2017). In humans, polyP participates in blood coagulation but is also involved in neurodegenerative diseases (Lempart and Jakob 2019).

In prokaryotes, two types of PPKs are known to catalyze the reversible formation of polyP from ATP: PPK1 type PPKs have a molecular mass of ≈ 80 kDa and consist of an N-terminal (N) domain, a head (H) domain, and two C-terminal domains (C1 and C2) (Ahn and Kornberg 1990; Zhu et al. 2005). The *E. coli* polyP kinase is the first described PPK and the prototype of type 1 PPKs (Ahn and Kornberg 1990). PPKs of the PPK2 type have roughly only half of the molecular masses of PPK1s (MW around 40 kDa) and are divided into three subtypes dependent on their substrate specificities for nucleoside di-phosphates (subtype 1), nucleoside mono-phosphates (subtype 2), or both (subtype 3) (Motomura et al. 2014). Bacteria can have only the PPK1 type of PPKs, only the PPK2 type of PPKs, or both types of PPKs (PPK1 and PPK2) (Rao et al. 2009). Several recent reports describe that some PPK2s are able to form nucleotides with four (Mordhorst et al. 2019; Ogawa et al. 2019; Hildenbrand et al. 2019) or even five (Mordhorst et al. 2019) phosphate residues.

The β-proteobacterium *Ralstonia eutropha* (*Cupriavidus necator*) is somehow special as its genome has seven *ppk* genes. Indeed, two PPK1s (PPK1a and PPK1b) as well as five PPK2s (PPK2a–PPK2d) have been identified in *R. eutropha* (Tumlirsch et al. 2015). One of the PPK2s, PPK2c, turned out to be highly active in vitro and catalyzed the formation of microscopically detectable polyP granules in vitro (Hildenbrand et al. 2019; Hildenbrand et al. 2020). The other PPKs of *R. eutropha* have not yet been investigated. The α-proteobacterium *Agrobacterium tumefaciens* has two *ppk* genes (Atu0418 and Atu1144) that encode a type 2 PPK (PPK2_AT_) and a type 1 PPK (PPK1_AT_), respectively. In our recent study (Frank and Jendrossek 2020), we showed that polyP in *Agrobacterium tumefaciens* is stored in “ordinary” polyP granules and not in membrane-enclosed acidocalcisomes as previously assumed (Seufferheld et al. 2003). In the present study, we determined the in vitro properties of purified PPK1_AT_ and PPK2_AT_. It turned out that PPK2_AT_ is rather promiscuous with respect to its nucleotide specificity but had the so far unique property to catalyze the formation of oligophosphorylated nucleotides up to nona-phosphorylated nucleotides.

## Material and methods

### Bacterial strains, plasmids, and culture conditions

Table 1 lists all used bacterial strains and plasmids of this study. *A. tumefaciens* C58 was the source of *ppk* genes. Genomic DNA was used to clone *ppk1* (Atu1144, hereafter *ppk1*_*AT*_) and *ppk2* (Atu0418, hereafter *ppk2*_*AT*_). *Escherichia coli* JM109 and *E. coli* BL21(DE3)/pLysS (Novagen) served as hosts in cloning procedures and gene expression, respectively. Cloning of *ppk1*_*AT*_ into the pET22b expression vector (*ppk1*_*AT*_*-his*_*6*_) was done with *Bam*HI and *Sac*I as restriction and cloning sites. *Ppk2*_*AT*_ was cloned into pET28a (*his*_*6*_*-ppk2*_*AT*_) with *Nco*I and *Bam*HI. The pET22b-*ppk1*_*AT*_ and pET28a-*ppk2*_*AT*_ constructs were transformed into *E. coli* JM109, verified by PCR amplification and sequencing, before finally transformed into the expression strain *E. coli* BL21(DE3)/pLysS.

**Table 1:** Bacterial strains and plasmids used in this study

Strain/plasmid	Relevant characteristics	Reference
*Escherichia coli* JM109	Cloning strain	DSMZ3423
*E. coli* BL21(DE3)/pLysS		Novagene
*Agrobacterium tumefaciens* C58	Wild type, Km^s^	Goodner et al. (2001)
*A. tumefaciens *C58* Δppk1 Δppk2*	Gene deletion of *ppk1*_*AT*_ (*atu1144*) and *ppk2*_*AT*_ (*atu0418*), polyP-deficient, Km^s^	Frank and Jendrossek (2020)
pBBR1MCS2::P*phaC-eyfp-c1*	Broad host range vector for construction of gene fusions with *eyfp*, confers Km^r^, constitutive expression from P*phaC*	Pfeiffer et al. (2011)
pBBR1MCS2::P*phaC-mCherry-n1*	Broad host range vector for construction of gene fusions with *mCherry*, confers Km^r^, constitutive expression from P*phaC*	Pfeiffer et al. (2011)
pBBR1MCS2::P*PhaC-ppk1*_*AT*_*-mCherry*	Constitutive expression of *ppk1*_*AT*_-*mCherry* (*atu1144*)	This study
pBBR1MCS2::P*PhaC-eyfp-ppk1*_*AT*_	Constitutive expression of *eyfp-ppk2*_*AT*_	Frank and Jendrossek (2020)
*Ralstonia eutropha* H16	Wild type, Km^s^	DSMZ428
*R. eutropha *H16* Δppk-all*	Chromosomal deletion of *Δppk1a*, *Δppk1b*, *Δppk2a*, *Δppk2b*, *Δppk2c*, *Δppk2d*, *Δppk2e* (*A0104*, *B1019*, *A0226*, *A0997*, *A1212*, *A0997*, *A1271*, *A1979*), Km^s^ polyP deficient	Tumlirsch and Jendrossek (2017)

Kanamycin resistance (Km^r^) and sensitivity (Km^s^)

### Purification of PPK1_AT_ and PPK2_AT_ and PPK activity assays

Purifications of PPK1_AT_ with a C-terminal and PPK2_AT_ with an N-terminal hexa-histidine tag were done as previously described for PPK2c of *Ralstonia eutropha* (Hildenbrand et al. 2019). Purified PPK proteins (1–4 mg/ml) were stored on ice directly in elution buffer (pH 8.0 (composition see below) without any further treatment (250 mM imidazole, 300 mM NaCl and 50 mM NaH_2_PO_4_). PPK activity assays and reaction product determination via HPLC were performed as described in detail previously (Hildenbrand et al. 2020).

#### Reaction conditions for the formation of oligophosphorylated nucleosides by PPK2_AT_ and HPLC-MS/MS analysis

Samples containing 1 μM enzyme, 15 mM NTPs, 2 mM MnCl_2_, and 0.1 M Tris-HCl buffer (pH 7–8) were incubated for times as indicated (30 min up to 48 h at 30 °C). Alternatively, 1 μM PPK2_AT_ was incubated with 2 mM NDP (or dTDP [TDP not available]) and 9 mM polyP in assay buffer (0.1 M Tris-HCl Buffer, pH 8, 2 mM MnCl_2_) for times as indicated. The reactions were terminated by heating of the assay mixture to 95 °C for 3 min. For later use, the samples were stored at − 20 °C or measured directly**.** Samples were thawed on ice and centrifugated for 15 min at 20,000 *g* and 4 °C. Targeted LC-MS measurements were performed with enhanced sensitivity on an Agilent 1200 HPLC system coupled with an Agilent 6410B triple quadrupole mass spectrometer (QQQ). Sample preparation and chromatographic separation of nucleoside phosphates by bicratic polymer-based zwitterionic hydrophilic interaction chromatography (ZIC-pHILIC) were performed as previously described (Feith et al. 2019; Hildenbrand et al. 2020) with following modifications: Mobile phases (constant flow rate of 0.2 ml min^−1^) were composed of aqueous buffer solutions (10 mM ammonium acetate, pH 9.2) with 90% (v/v) acetonitrile for eluent A and 10% (v/v) acetonitrile for eluent B, using the following program for gradient elution: isocratic hold 0% B for 1 min, linear gradient from 0% B to 70% B for 19 min, linear gradient from 70% B to 100% B for 2 min, isocratic hold 100% B for 2.5 min, linear gradient from 100% B to 0% B for 4 min, and equilibration to starting conditions by an isocratic hold 0% B for 12 min. Nucleoside mono-, di-, and triphosphates were detected in negative ionization mode (ESI-) with high sensitivity in selected ion monitoring (SIM) mode based on pre-optimized precursor ion transitions with a mass resolution of 0.3 u and associated MS parameters (Teleki et al. 2015). Higher oligophosphorylated nucleosides were detected by calculated precursor ion transitions and transferred MS parameters. System control, acquisition, and data analysis were obtained by using commercial MassHunter B.07.00 software.

### Toluidine staining of polyP after polyacrylamide gel electrophoresis

Enzymatically produced polyP (by (PPK1_AT_ or PPK2_AT_) was separated by electrophoresis in 15% poly acrylamide gels and stained with toluidine blue according to (Losito et al. 2009). A synthetic polyP standard with ≈ 100 P_i_ residues in average (gift of A. Saidari) was used as standard. PolyP concentrations in this contribution always refer to the concentration of the monomeric phosphate (P_i_).

### PolyP quantification

The amount of polyP was quantified after polyP extraction followed by digestion to phosphate with exopolyphosphatase and subsequent phosphate determination (malachinte green assay) as recently summarized in (Christ et al. 2020b).

## Results

### Purification of polyP kinases

The two polyP kinase genes of *A. tumefaciens* (*ppk1*_*AT*_) and (*ppk2*_*AT*_) were PCR amplified, cloned in fusion with a hexa-histidine-coding sequence into pET22b or pET28a, respectively, and expressed in *E. coli* BL21. PPK1_AT_ and PPK2_AT_ eluted during nickel agarose affinity chromatography of soluble cell extracts at approximately 250 mM imidazole. PPK1_AT_ and PPK2_AT_ containing fractions were combined, concentrated, and tested for purity on a polyacrylamide gel (Online resource 1). PPK1_AT_ (84.9 kDa) was strongly enriched and PPK2_AT_ (36.2 kDa) was almost homogeneous.

### Biochemical characterization of PPK1_AT_

The substrate specificity of PPK1_AT_ was analyzed by incubation of PPK1_AT_ with ATP, GTP, CTP, dTTP, or UTP for 30 min and subsequent determination of the respective nucleotide compositions by HPLC. It turned out that the activity of PPK1_AT_ with NTPs was hardly detectable if low concentrations of NTPs (2 mM) were used. Only at high concentrations of the NTPs (15 mM) and after 24 h reaction time polyP-forming activity was detected as revealed by formation of NDPs at the expense of NTPs (Online resource 2). The highest activity was determined with the purines ATP and GTP. With GTP as substrate (but not with other NTPs), the formation of polyP was shown by gel electrophoresis and subsequent staining with toluidine (Online resource 3). Presumably, the concentration of formed polyP by the other nucleotides was too low to be detected by toluidine staining.

**Fig. 1:**
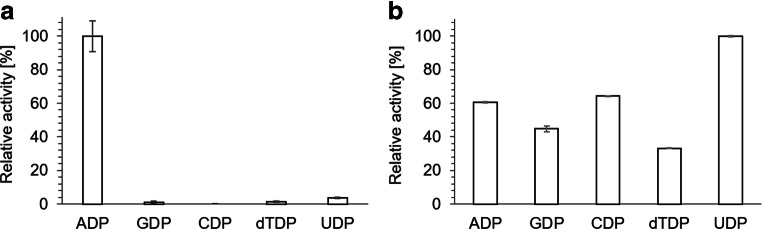
Enzyme activity of PPK1_AT_. The reactions were performed in the direction of NTP synthesis from NDPs (2 mM) and polyP (9 mM P_i_) and in the presence of 2 mM MnCl_2_. **a** Relative activity (%) after 30-min incubation. The highest activity (0.004 μmol ADP/min/mg expressed as average of the 30-min incubation time) was determined with ADP + polyP and was taken as 100%. **b** Relative activity (%) after 6-h incubation. Assays were performed in triplicates; error bars indicate standard deviations

**Fig. 2:**
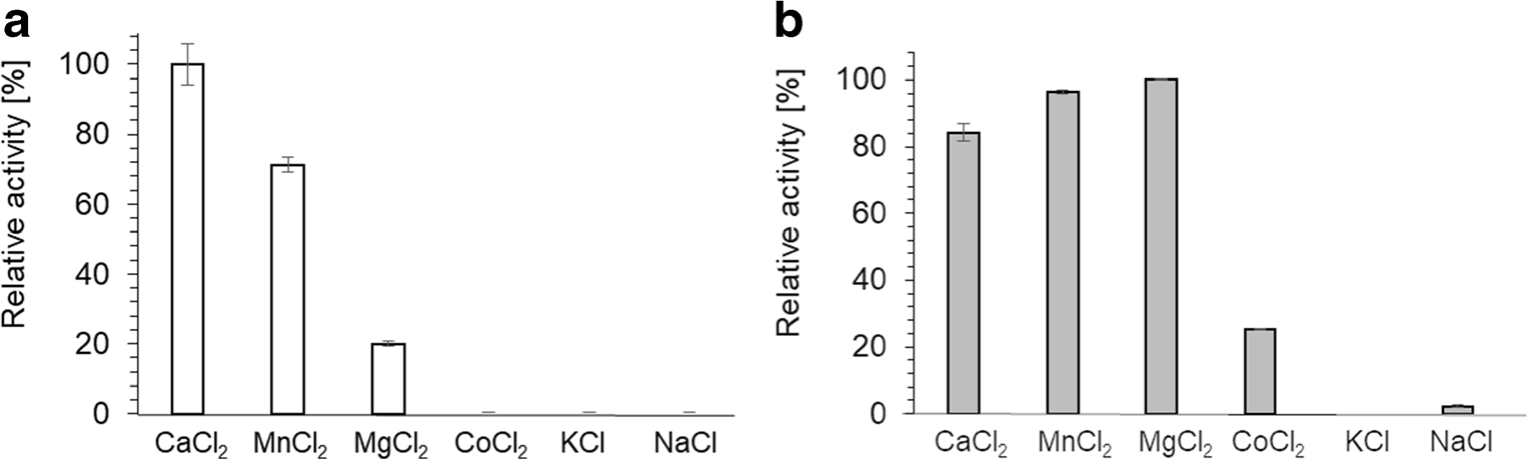
Enzyme activities of PPK1_AT_ and PPK2_AT_ with different cations. Activities of PPK1_AT_ (**a**) in polyP synthesis direction (from 2 mM ATP, 30 min reaction time) and of PPK2_AT_ (**b**) in NTP synthesis direction (from 2 mM ADP, 9 mM polyP, 30-min reaction time) with different cations (2 mM) were determined. The activities were maximal with CaCl_2_ (PPK1_AT_, 0.01 μmol ATP/min/mg expressed as average of the first 30 min) or with MgCl_2_ (PPK2_AT_, 0.09 μmol ADP/min/mg) expressed as average of the first 30 min), respectively, and were taken as 100%. Assays were performed in triplicate; error bars indicate standard deviations

When PPK1_AT_ activity was tested in the direction of NTP synthesis from polyP and NDPs, an activity of 0.037 μmol formed ATP/min/mg (using the 5-min incubation time) was determined for the conversion of ADP (Fig. 1a) but hardly any NTP-synthesizing activities were detected for the other nucleotides (GDP, CDP, UDP, or dTDP). Only by elongation of the assay time to 6 h the formation of other NTPs from the respective NDPs and polyP became detectable (Fig. 1b). The formation of adenosine tetraphosphate (AP4), however, was not detected (data not shown). AP4 and related NP4s had been previously detected as reaction products of PPKs in earlier studies (see “Discussion” for details and references). PPK1_AT_ showed good activity in the presence of either calcium or manganese as cofactor. With magnesium, the activity was surprisingly low and no activity was determined in the absence of divalent cations (Fig. 2a). No kinase activity was detected for purified PPK1_AT_ with any nucleoside monophosphate and polyP up to 24 h incubation (data not shown).

### Biochemical characterization of PPK2_AT_

Purified PPK2_AT_ (as PPK1_AT_) was not able to phosphorylate NMPs with polyP as phosphate donor (data not shown) and therefore is a member of subgroup 1 of PPK2s according to the PPK2 classification of Motomura et al. (Motomura et al. 2014).

**Fig. 3:**
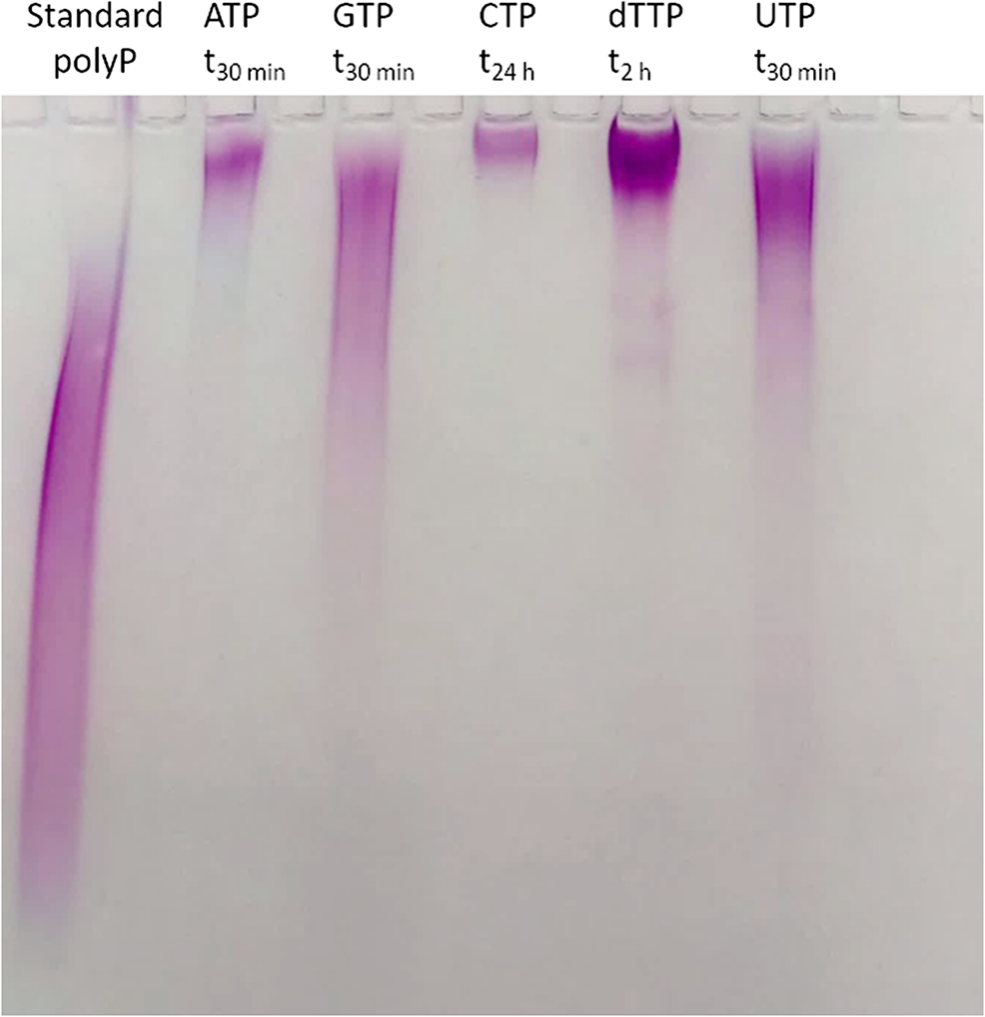
Separation of PPK2_AT_-produced polyP by PAGE and subsequent toluidine staining. PolyP (average chain length ≈ 100 P_i_ residues corresponding to 9 mM P_i_) (lane 1), products from15 mM of ATP after 30 min (lane 3), GTP after 30 min (lane 5), CTP after 24 h (lane 7), dTTP after 2 h (lane 9) or UTP after 30 min (lane 11), and controls with no NTP (lane 2) or without PPK2_AT_ (lane 13). Note the products were obtained after different assay times. For direct comparison of products after 30 min and 24 h, see Online resource 5a and 5b

PPK2_AT_ catalyzed the formation of polyP from ATP (NTP) as evident from the decrease of the ATP (NTP) concentration and a concomitant increase of the ADP (NDP) concentrations in PPK assays with purified PPK2_AT_ after an incubation period of 30 min (Online resource 4). When the reaction products were gel-electrophoretically separated and stained with toluidine blue, strong staining signals were detected as a pink smear and indicated that a mixture of long-chain polyP molecules had been formed (Fig. 3, Online resource 5a). In these experiments, Mn^2+^ ions were used as cofactor. However, Mn^2+^ could be replaced by Mg^2+^ and by Ca^2+^ but not by monovalent ions such as Na^+^ or K^+^. Poor activity of PPK2_AT_ was detected in the presence of Co^2+^ (Fig. 2b).

Similar results were obtained when PPK2_AT_ was incubated with GTP or UTP (instead of ATP) but not with CTP or dTTP as substrates for 30 min. However, after longer incubation time, polyP-toluidine signals became detectable also for dTTP (2 h) and for CTP (24 h) (Fig. 3, Online resource 5b). Interestingly, the polyP-toluidine signals that were clearly detectable after 30 min for GTP, dTTP (2 h), and UTP as substrates vanished after longer incubation times; instead, nucleoside phosphates with more than three phosphate residues were detected by HPLC from GTP, dTTP, and UTP (see next chapter for details). This indicated that polyP was used as an intermediate substrate for the synthesis of guanosine-, d-thymidine-, or uridine-oligophosphates in case of the reactions with GTP, dTTP, and UTP. In contrast, even after 48 h of incubation, the polyP-toluidine signals produced from ATP and CTP were still present and no oligophosphorylated nucleotides (except NP4) were detected with ATP and CTP by HPLC and UV-vis detection (for overview see Online resource 6).

### PPK2_AT_ phosphorylates NDPs with polyP as phosphor donor and produces NTPs and nucleoside oligophosphates

**Fig. 4:**
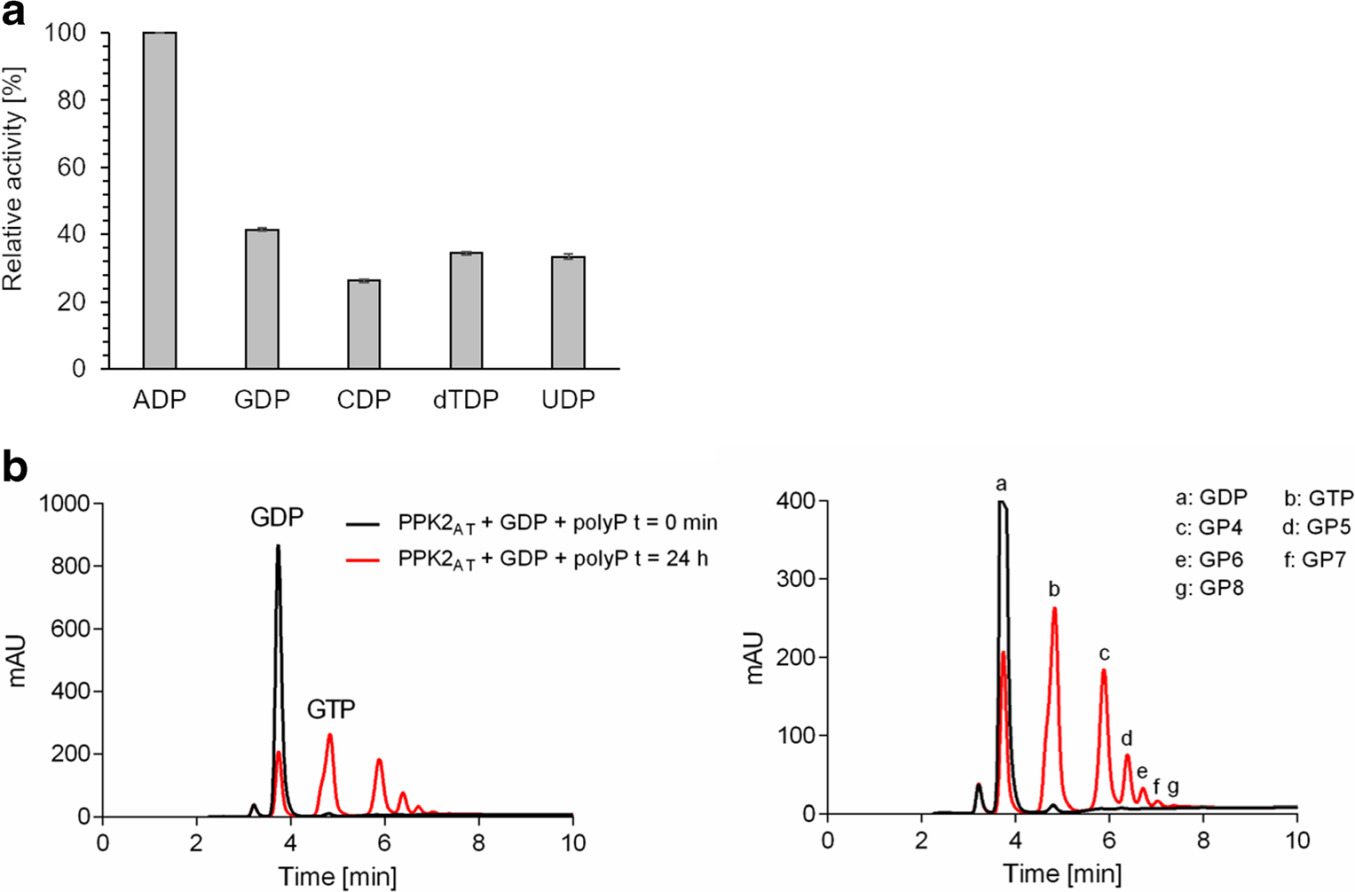
**a** Substrate specificity of PPK2_AT_. PPK2_AT_ was assayed in the direction of NTP synthesis from NDP (2 mM) and polyP (9 mM as P_i_) with MnCl_2_ as cofactor for 30 min. Highest activity was determined for ADP and polyP (0.097 μmol ADP/min/mg) and was taken as 100%. Assays were performed in triplicate; error bars indicate standard deviations. **b** Formation of oligophosphorylated nucleosides by PPK2_AT_. Educts and products of PPK2_AT_-catalyzed reactions after 24 h of incubation of GDP and polyP were analyzed by HPLC. The left image shows the concentrations of guanosine nucleotides before (0 min, black) and after the addition of PPK2_AT_ (24 h, red, mAU, milli-absorption units at 254 nm). The image on the right is an enlargement revealing the presence of several additional (minor) peaks that correspond to oligophosphorylated guanosine nucleosides (GP4–GP8)

The identification of HPLC signals after prolonged incubation of some NTPs with PPK2_AT_ that most likely corresponded to oligophosphorylated nucleosides prompted us to investigate whether these assumed oligophosphorylated nucleosides were also formed from NDPs in the presence of polyP. To this end, purified PPK2_AT_ was incubated with ADP, GDP, CDP, dTDP, or UDP (each 2 mM) in the presence of 9 mM polyP for 24 h. Analysis of the products by HPLC revealed that the concentrations of the respective NDPs were substantially reduced (Fig. 4a) and the concentrations the corresponding NTPs were increased. Additional peaks appeared in the HPLC chromatograms of all tested NDPs at higher retention time than that of the respective NTP in each assay and corresponded to adenosine tetraphosphate (AP4), guanosine tetraphosphate (GP4), cytosine tetraphosphate (CP4), desoxy-thymidine tetraphosphate (dTP4), and uridine tetraphosphate (UP4) (Online resource 6) as it has been previously shown for other PPK2s. Apparently, PPK2_AT_ is a member of the growing group of PPK2s that catalyzes the formation of nucleoside tetraphosphates from NDPs and polyP (see below for more details and references). Furthermore, the HPLC chromatograms of the PPK2_AT_ assays showed even more additional peaks at higher retention times than the respective NP4s for the reactions with GDP, CDP, dTDP, and UDP (exemplarily shown for the reaction with GDP and polyP in Fig. 4b, all other chromatograms in Online resource 6). Assuming that each peak at higher retention time than the respective NTP corresponds to molecules with one more phosphate group, this would mean that PPK2_AT_ can catalyze the formation of guanosine oligophosphates (GP4-GP8), cytosine oligophosphates (CP4-CP8), desoxy-thymidine oligophosphates (TP4-TP8), and uridine oligophosphates (UP4-UP7). The retention times of these additional peaks were identical to the retention times of the additional peaks that had been detected after prolonged incubation of PPK2_AT_ with GTP, dTTP, or UTP (see above). We assume that these peaks represent nucleotides with a higher number of phosphate residues than four (see Online resource 7 for summary). Nucleotides with four and five phosphate residues have been previously described as reaction products of PPKs (Mordhorst et al. 2019) but higher phosphorylated nucleosides have not yet been described.

### Identification of nucleoside oligophosphates by ZIC-pHILIC-MS/MS

**Fig. 5:**
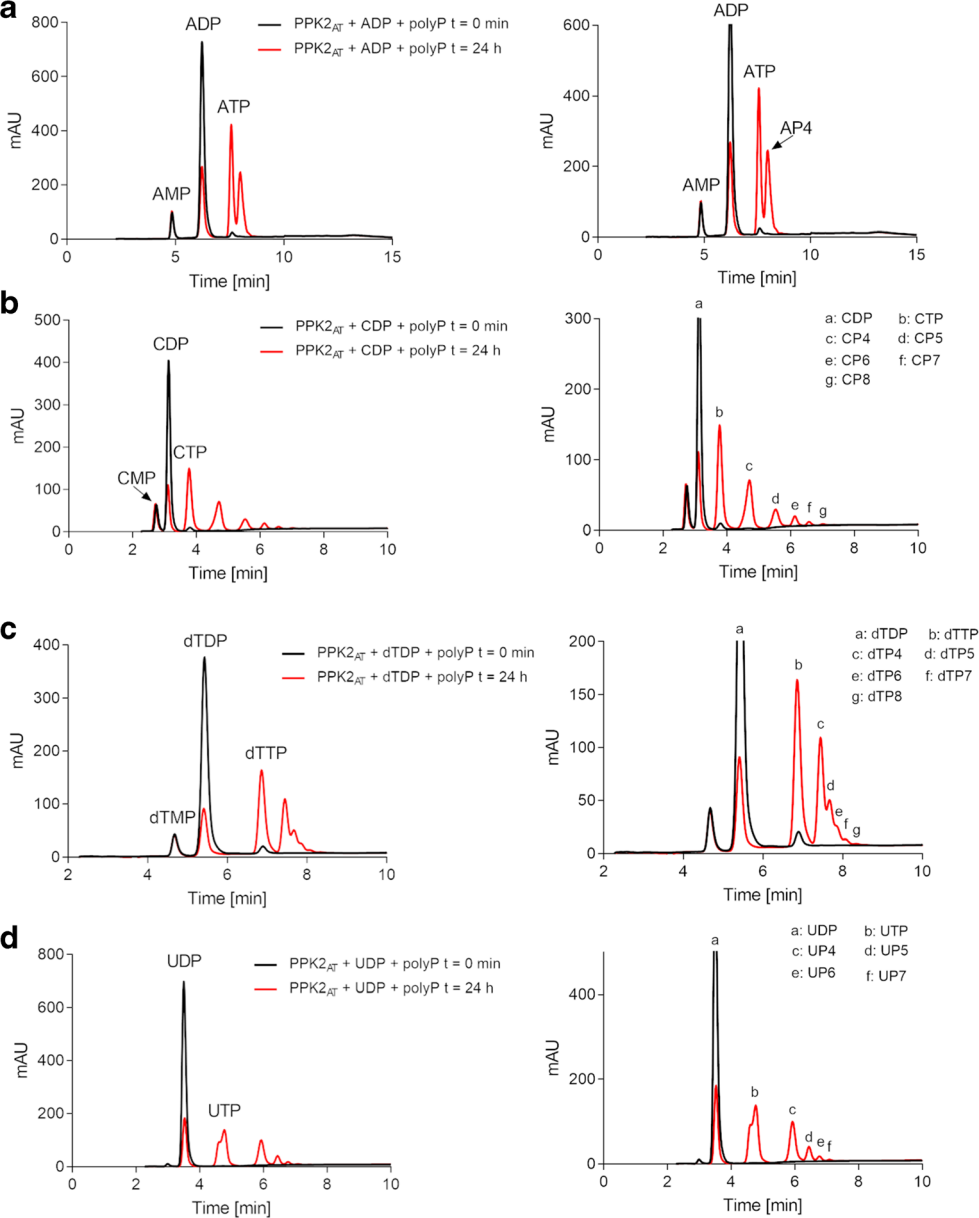
Formation of oligophosphorylated nucleosides by PPK2_AT_. The reaction products of PPK2_AT_ after 24 h of incubation with NDPs in the presence of polyP were analyzed by HPLC. The left image shows the concentrations of nucleotides before (0 min, black) and after the addition of PPK2_AT_ (24 h, red, mAU, milli-absorption units at 254 nm). In the enlargement in the image on the right, several additional (minor) peaks became detectable that correspond to oligophosphorylated nucleotides

To verify that the observed products really represent the postulated oligophosphorylated nucleosides, the products of the PPK2_AT_-catalyzed reaction with NTPs were analyzed by HPLC and HPLC-MS/MS. Figure 5 shows the products of the reaction of PPK2_AT_ with NTPs separated by HPLC. Again, peaks appeared at higher retention times than the corresponding NTPs and were identified as oligophosphorylated nucleotides by ZIC-pHILIC-MS/MS. Notably, the deduced masses perfectly matched the expected masses of guanosine tetra-, penta-, hexa-, hepta-, and octa-phosphate each with mass increments of 80 (corresponding to one phosphate unit in polyP; see Table 2). The MS detector even detected molecules with *m/z* values that corresponded to guanosine nona-phosphate (GP9) and guanosine deca-phosphate (GP10), however, with insufficient signal-to-noise ratios. The signal-to-noise ratios were too low for GP9 and GP10 to allow a reliable identification and the concentrations of the higher phosphorylated nucleosides were too low to be visible in the HPLC UV-vis detection. Analog results were obtained for the products of PPK2_AT_ with the other nucleotides (ATP, GTP, CTP, dTTP, and UTP). With each nucleotide, *m/z* signals for up to the octa-phosphorylated nucleosides (and up to the nona-phosphorylated nucleotide in case of ATP) were clearly detected by ZIC-pHILIC-MS/MS. For a summary of all identified nucleotides, see Table 2 and Online resource 7 and 8. In summary, PPK2_AT_ catalyzes the transfer of multiple P_i_ residues from polyP to NDPs with up to the nona-phosphorylated nucleosides as products. Presumably, nucleotides with even more phosphate residues than eight will become detectable by variation of the assay parameters and/or optimization of the MS detection parameters.

**Table 2:** Results and parameters of HILIC-QQQ-MS/MS measurements of nucleotide species in PPK2_AT_ assays and standard mixtures

Nucleotides		MS/MS parameters (SIM)			HILIC-QQQ-SIM results				HPLC results
Abbr.	Sum formula	ESI	Precursor		PPK2_AT_ assay		100 μM standard mix		PPK2_AT_ assay
		Polarity	Species	*m/z* (u)	Area (Cts)	*t*_R_* (min)	Area (Cts)	*t*_R_* (min)	*t*_R_* (min)
GMP	C_10_H_14_N_5_O_8_P	[-]	[M-H]^-^	362	1.89E+07	17.63	6.82E+07	17.59	3.1
GDP	C_10_H_15_N_5_O_11_P_2_	[-]	[M-H]^-^	442	6.43E+08	19.27	1.73E+08	19.33	3.3
GTP	C_10_H_16_N_5_O_14_P_3_	[-]	[M-H]^-^	522	4.64E+08	20.64	1.58E+08	20.76	4.0
GP4	C_10_H_17_N_5_O_17_P_4_	[-]	[M-H]^-^	602	2.39E+08	22.16			4.5
GP5	C_10_H_18_N_5_O_20_P_5_	[-]	[M-H]^-^	682	3.55E+07	23.82			5.2
GP6	C_10_H_19_N_5_O_23_P_6_	[-]	[M-H]^-^	762	5.49E+06	25.32			5.8
GP7	C_10_H_20_N_5_O_26_P_7_	[-]	[M-H]^-^	842	5.84E+05	26.84			6.3
GP8	C_10_H_21_N_5_O_29_P_8_	[-]	[M-H]^-^	922	7.31E+04	27.97			6.7
dTMP	C_10_H_15_N_2_O_8_P	[-]	[M-H]^-^	321	< LOD**	na***	1.66E+08	14.10	4.6
dTDP	C_10_H_16_N_2_O_11_P_2_	[-]	[M-H]^-^	401	6.76E+05	16.36	2.47E+08	16.35	5.5
dTTP	C_10_H_17_N_2_O_14_P_3_	[-]	[M-H]^-^	481	2.21E+07	18.04	1.84E+08	17.86	6.1
dTP4	C_10_H_18_N_2_O_17_P_4_	[-]	[M-H]^-^	561	4.94E+08	20.89			6.9
dTP5	C_10_H_19_N_2_O_20_P_5_	[-]	[M-H]^-^	641	1.08E+08	22.58			7.3
dTP6	C_10_H_20_N_2_O_23_P_6_	[-]	[M-H]-	721	5.99E+07	24.10			7.5
dTP7	C_10_H_21_N_2_O_26_P_7_	[-]	[M-H]^-^	801	2.03E+07	25.49			7.7
dTP8	C_10_H_22_N_2_O_29_P_8_	[-]	[M-H]^-^	881	7.33E+06	26.76			7.9
AMP	C_10_H_14_N_5_O_7_P	[-]	[M-H]^-^	346	1.67E+08	15.09	1.36E+08	15.09	4.7
ADP	C_10_H_15_N_5_O_10_P_2_	[-]	[M-H]^-^	426	4.63E+08	16.69	1.76E+08	16.90	6.4
ATP	C_10_H_16_N_5_O_13_P_3_	[-]	[M-H]^-^	506	1.02E+09	18.44	1.85E+08	18.32	7.8
AP4	C_10_H_17_N_5_O_16_P_4_	[-]	[M-H]^-^	586	2.75E+08	19.96			8.2
AP5	C_10_H_18_N_5_O_19_P_5_	[-]	[M-H]^-^	666	1.60E+08	22.26			na
AP6	C_10_H_19_N_5_O_22_P_6_	[-]	[M-H]^-^	746	8.38E+06	23.61			na
AP7	C_10_H_20_N_5_O_25_P_7_	[-]	[M-H]^-^	826	2.08E+06	24.82			na
AP8	C_10_H_21_N_5_O_28_P_8_	[-]	[M-H]^-^	906	3.22E+05	26.10			na
AP9	C_10_H_22_N_5_O_31_P_9_	[-]	[M-H]^-^	986	1.47E+05	27.27			na
UMP	C_9_H_13_N_2_O_9_P	[-]	[M-H]^-^	323	1.59E+06	16.23	1.08E+08	16.23	2.9
UDP	C_9_H_14_N_2_O_12_P_2_	[-]	[M-H]^-^	403	2.27E+07	17.92	1.98E+08	18.15	3.1
UTP	C_9_H_15_N_2_O_15_P_3_	[-]	[M-H]^-^	483	1.69E+07	19.27	2.35E+08	19.57	3.8
UP4	C_9_H_16_N_2_O_18_P_4_	[-]	[M-H]^-^	563	1.27E+07	20.98			4.4
UP5	C_9_H_17_N_2_O_21_P_5_	[-]	[M-H]^-^	643	1.91E+06	22.56			5.2
UP6	C_9_H_18_N_2_O_24_P_6_	[-]	[M-H]^-^	723	2.16E+05	24.10			5.9
UP7	C_9_H_19_N_2_O_27_P_7_	[-]	[M-H]^-^	803	5.75E+03	26.04			6.4
UP8	C_9_H_20_N_2_O_30_P_8_	[-]	[M-H]^-^	883	4.78E+03	27.12			6.8
CMP	C_9_H_14_N_3_O_8_P	[-]	[M-H]^-^	322	3.36E+07	16.90	8.69E+07	16.95	2.6
CDP	C_9_H_15_N_3_O_11_P_2_	[-]	[M-H]^-^	402	6.04E+08	18.42	1.35E+08	18.63	3.0
CTP	C_9_H_16_N_3_O_14_P_3_	[-]	[M-H]^-^	482	6.97E+08	19.89	1.21E+08	20.00	3.7
CP4	C_9_H_17_N_3_O_17_P_4_	[-]	[M-H]^-^	562	7.24E+07	21.64			na
CP5	C_9_H_18_N_3_O_20_P_5_	[-]	[M-H]^-^	642	1.50E+06	23.25			na
CP6	C_9_H_19_N_3_O_23_P_6_	[-]	[M-H]^-^	722	4.64E+04	24.78			na
CP7	C_9_H_20_N_3_O_26_P_7_	[-]	[M-H]^-^	802	< LOD**	na			na
CP8	C_9_H_21_N_3_O_29_P_8_	[-]	[M-H]^-^	882	< LOD**	na			na

*Retention time of the nucleotide species

**Limit of detection

***Not available

Peak areas (Cts) and HILIC retention times (min) were achieved by QQQ-SIM detection (*m/z* ± 0.3 u). Retention times of HPLC measurements (min) are comparatively depicted in the most right column

#### Expression of *ppk1*_*AT*_ and *ppk2*_*AT*_ in polyP-deficient backgrounds

**Fig. 6:**
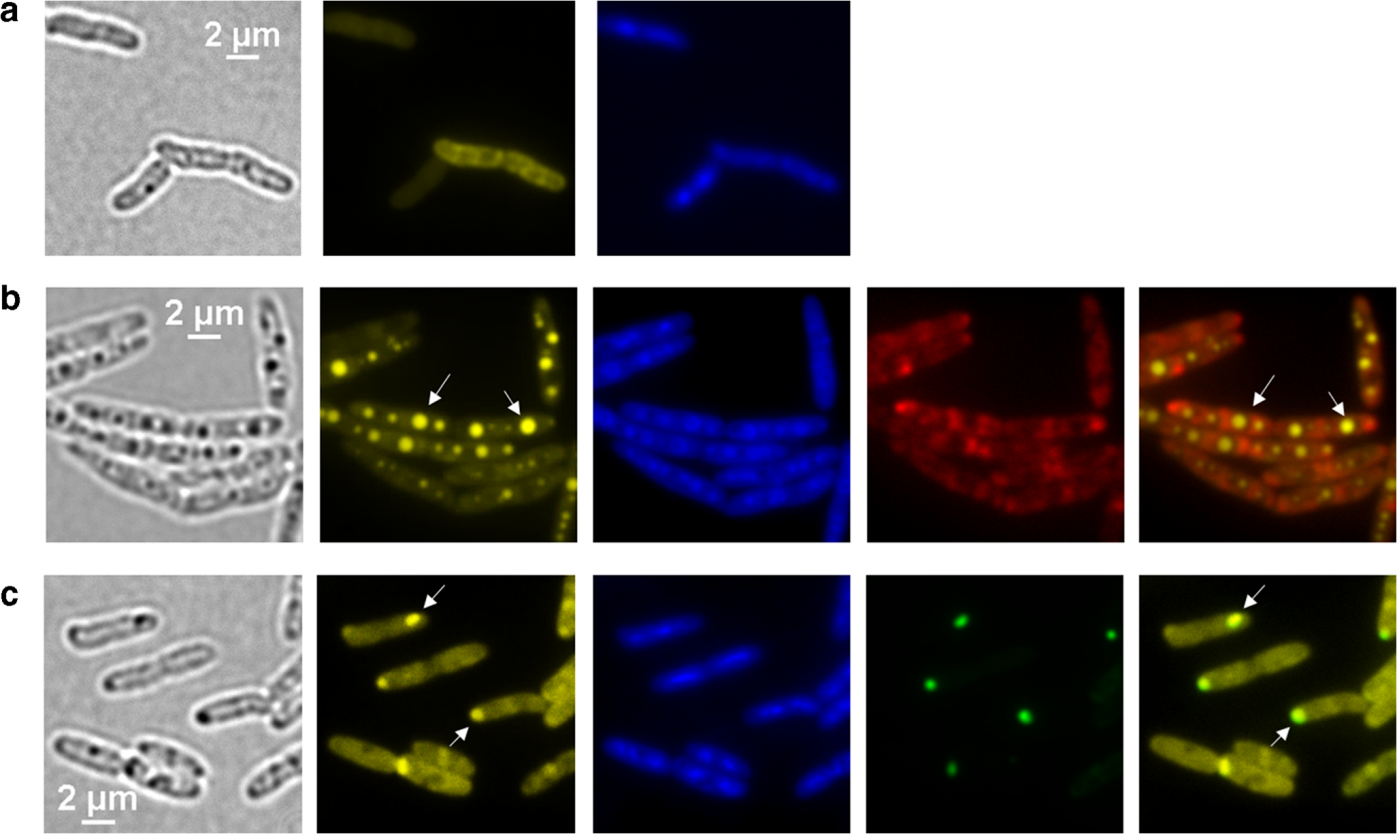
Expression of *ppk1*_*AT*_ and *ppk2*_*AT*_ in *R. eutropha*. NB-grown cells were stained with DAPI and imaged in bright-field and fluorescence microscopy as indicated. **a** *R. eutropha ∆ppk-all* (polyP-deficient). From left to right: bright field, DAPI-polyP channel and DAPI-DNA channel. **b** *R. eutropha ∆ppk-all* harboring pBBR1MCS2::P*phaC-ppk1*_*AT*_*-mCherry* shows various DAPI-stainable polyP granules distributed over the length of the cell, whereas the mCherry-PPK1_AT_ signal is located throughout the cytoplasm. From left to right: bright field, DAPI-polyP channel, DAPI-DNA channel, mCherry channel, merge of DAPI-polyP, and mCherry channel. **c** *R. eutropha ∆ppk-all* harboring pBBR1MCS2::P*phaC-eyfp*-*ppk2*_*AT*_*.* The cells were able to form DAPI-stainable polyP granules at the cell pole that colocalize with the eYFP-PPK2_*AT*_ fluorescence signal

To test the function of the *A. tumefaciens ppk* genes in vivo, we expressed the *ppk1*_*AT*_ and *ppk2*_*AT*_ genes as fusions with the enhanced yellow fluorescent gene (*eyfp*) (or with *mCherry*) in a polyP-deficient background of *A. tumefaciens* (∆*ppk1*_*AT*_, *ppk2*_*AT*_) and *R. eutropha* (*∆ppk-all*, with all seven *ppk* genes deleted). Expression of *ppk1*_*AT*_ restored the formation of polyP granules in *A. tumefaciens* (∆*ppk1*_*AT*_, *ppk2*_*AT*_) (Online resource 9) and confirmed that PPK1_AT_ is able to form polyP in vivo. A similar result was obtained when *ppk1*_*AT*_ was expressed in *R. eutropha ∆ppk-all* (Fig. 6b). To our surprise, the expression of *ppk2*_*AT*_ also resulted in restoration of polyP granule synthesis in the polyP-deficient *R. eutropha* background (Fig. 6c) but not in *A. tumefaciens* (∆*ppk1*_*AT*_, *ppk2*_*AT*_) (Online resource 9). These results confirmed that both PPKs of *A. tumefaciens* principally are able to catalyze the formation of polyP in vivo. In most cells, the formed fusion proteins (eYFP-PPK2_AT_) colocalized with DAPI-stained polyP granules in both species. However, PPK1_AT_-mCherry signals were elliptically shaped and were located near the cell pole in *A. tumefaciens* sometimes—but not always—in close proximity to a formed polyP granule (Online resource 9). In *R. eutropha Δppk-all*, the same construct was distributed more or less homogenously in the whole cell around the polyP granules (Fig. 6b), but did not colocalize with them.

## Discussion

This activity and substrate specificity determinations in our study showed that PPK1_AT_ is a typical type 1 PPK (Rao et al. 2009) having a preference (or high affinity) for adenine nucleotides and that PPK2_AT_ is a member of the subtype 1 group of PPK2s with specificity for NDPs but not for NMPs as it had been previously postulated by Motomura et al. (2014) from amino acid sequence analysis. PPK2_AT_ has the so far unique property to transfer multiple phosphate groups to GDP, dTDP, or UDP (and to lower extent also to ADP and CDP) and to form oligophosphorylated products up to the octa-phosphorylated nucleosides or up to AP9 in case of adenine nucleotides. So far, only tetra- or penta-phosphorylated nucleotides have been detected as reaction products of PPK2 of *Meiococcus ruber* (Mordhorst et al. 2019). Using *A. tumefaciens* PPK2_AT_, the oligophosphorylated nucleosides were formed from the diphosphates in the presence of polyP immediately. In contrast, it took much longer until oligophosphorylated nucleosides became detectable if PPK2_AT_ was tested in the direction of polyP synthesis from NTPs: it was first necessary that polyP molecules were formed before up to the deca-phosphorylated nucleosides became detectable. This finding suggests that PPK2_AT_ catalyzes the transfer of short phosphate chains from polyP to NDPs. A multiple transfer of single phosphate residues from polyP to NDPs is also possible. The reaction worked best with GDP, dTDP, or UDP and with lower efficiency also with ADP (up to AP9) or with CDP (up to CP8). We do not know whether the in vitro detected oligophosphorylated nucleosides were also formed in vivo and if they were formed, what physiological function they might fulfill. The in vitro formation of oligophosphorylated nucleosides required the presence of Mn^2+^ ions that are not likely to be present in millimolar concentrations in vivo. Interestingly, in *Myxococcus xanthus*, AP4 (and diadenosine tetraphosphate and diadenosine penta-phosphate) have been detected and are formed by the action of some amino acyl tRNA synthetases and by phosphoglycerate kinase (Kimura et al. 2018) suggesting that an astonishing variety of oligophosphorylated nucleotides can be formed in microorganisms in vivo.

PPK2_AT_ is a rather unspecific enzyme and converts all tested NDPs to the corresponding NTPs as the main product in the presence of polyP at high rates. This suggests that the in vivo function of PPK2_AT_ is to replenish lowered NTP (and/or dNTP) pools from previously accumulated polyP during phases of enhanced demand, e.g., during DNA replication or at high transcription rates. This corresponds with previous studies in our lab in which we showed that PPK2_AT_ is not necessary for polyP formation and that *∆ppk1*_*AT*_ (Frank and Jendrossek 2020) deletion strains were not able to form polyP granules. Accordingly, a *∆ppk2*_*AT*_ deletion strain produced slightly more polyP than the wild type presumably because the polyP-consuming activity of PPK2_AT_ is absent. The reason why PPK2_AT_ was able to restore the formation of polyP granules in *R. eutropha* but not in *A. tumefaciens* (despite good in vitro polyP-forming activities of PPK2_AT_) might lie in different in vivo nucleotide concentrations in both strains. Higher in vivo concentrations of NTPs in *R. eutropha* compared with *A. tumefaciens* might allow the formation of polyP from expressed *ppk2*_*AT*_.

## Electronic supplementary material

ESM 1 (PDF 5727 kb)
