# Supplementary material for: Characterization of Agrobacterium tumefaciens PPKs reveals the formation of oligophosphorylated products up to nucleoside nona-phosphates
Source: Appl Microbiol Biotechnol. 2020 Oct 6;104(22):9683–92. doi: 10.1007/s00253-020-10891-7 (PMC7595981; doi:10.1007/s00253-020-10891-7)
Supplement: Supplementary file 1 — (PDF 5727 kb) [file 253_2020_10891_MOESM1_ESM.pdf]

Applied Microbiology and Biotechnology

Online Resources

Characterization of *Agrobacterium tumefaciens* PPKs reveal the formation of oligo-phosphorylated products up to nucleoside nona-phosphates

Celina Frank[1], Attila Teleki[2], and Dieter Jendrossek[1*]

[1]*Institute of Microbiology, University of Stuttgart, Germany*

[2]*Institute of Biochemical Engineering, University of Stuttgart, Germany*

*Correspondent footnote: Dieter Jendrossek
Institut für Mikrobiologie
Universität Stuttgart
Allmandring 31
70569 Stuttgart
Germany
Tel.: +49-711-685-65483
Fax: +49-711-685-65725
Email: dieter.jendrossek@imb.uni-stuttgart.de

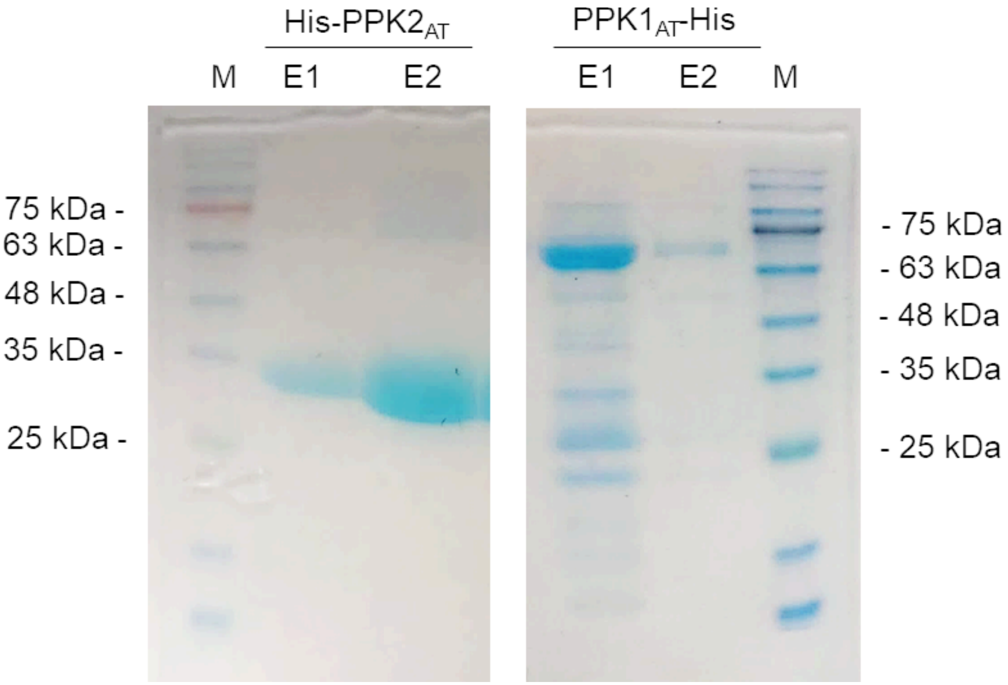

Online Resource 1: 12% SDS-polyacrylamide gel of $PPK2_{AT}$ (left) and $PPK1_{AT}$ (right) stained with colloidal Coomassie. M, marker proteins; E1, E2, elution steps. $PPK2_{AT}$ was homogeneous, while the $PPK1_{AT}$ preparation still had some contaminating proteins.

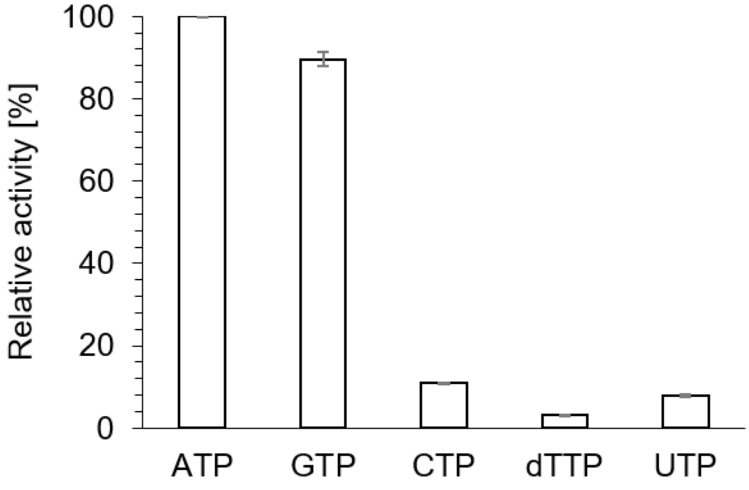

Online Resource 2: Relative activity [%] of $PPK1_{AT}$ after 24h incubation in the presence of 15 mM NTPs. The highest activity was determined with ATP and amounted 0.004 µmol /min/mg. Assays were performed in triplicates; error bars indicate standard deviations.

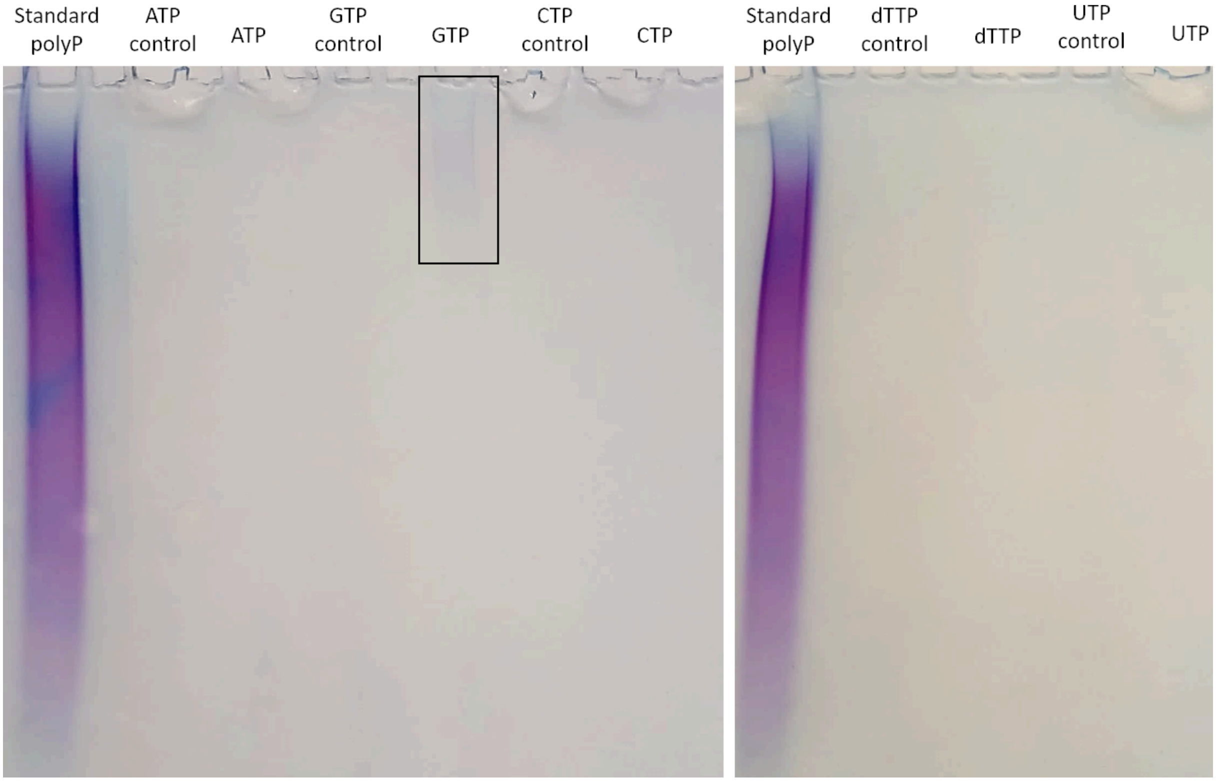

Online Resource 3: Separation of $PPK1_{AT}$-produced polyP after 24h by PAGE and subsequent toluidine staining. Standard polyP (average chain length ≈100 P_i residues corresponding to 9 mM P_i) (lanes 1 and 8). 15 mM of ATP, GTP, CTP, dTTP or UTP (lanes as indicated), controls with 15 mM ATP, GTP, CTP, dTTP or UTP but without $PPK2_{AT}$ (lanes as indicated). The black box indicates polyP product formed by the action of $PPK1_{AT}$ from GTP.

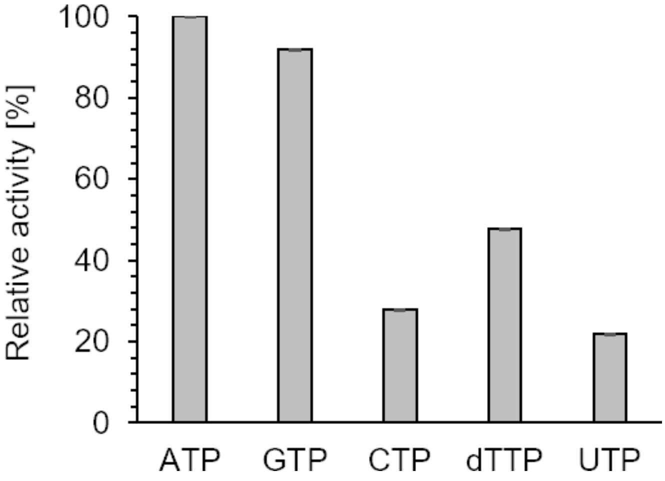

Online Resource 4: Nucleotide specificity of $PPK2_{AT}$. NTPs as indicated (15 mM) were incubated with purified $PPK2_{AT}$ for 30min in the presence of 2 mM $MnCl_2$ and the concentrations of the formed NDPs were determined by HPLC. Assays were performed in triplicates; error bars indicate standard deviations. The highest activity was determined with 2 mM ATP in the first 40 s of the reaction and amounted 0.09 µmol ATP/min/mg (data not shown).
For comparison of the activities of $PPK2_{AT}$ with different nucleotides (15 mM), the activity averaged over 30 min incubation time (2.8 µmol ATP/min/min) was taken as 100%.

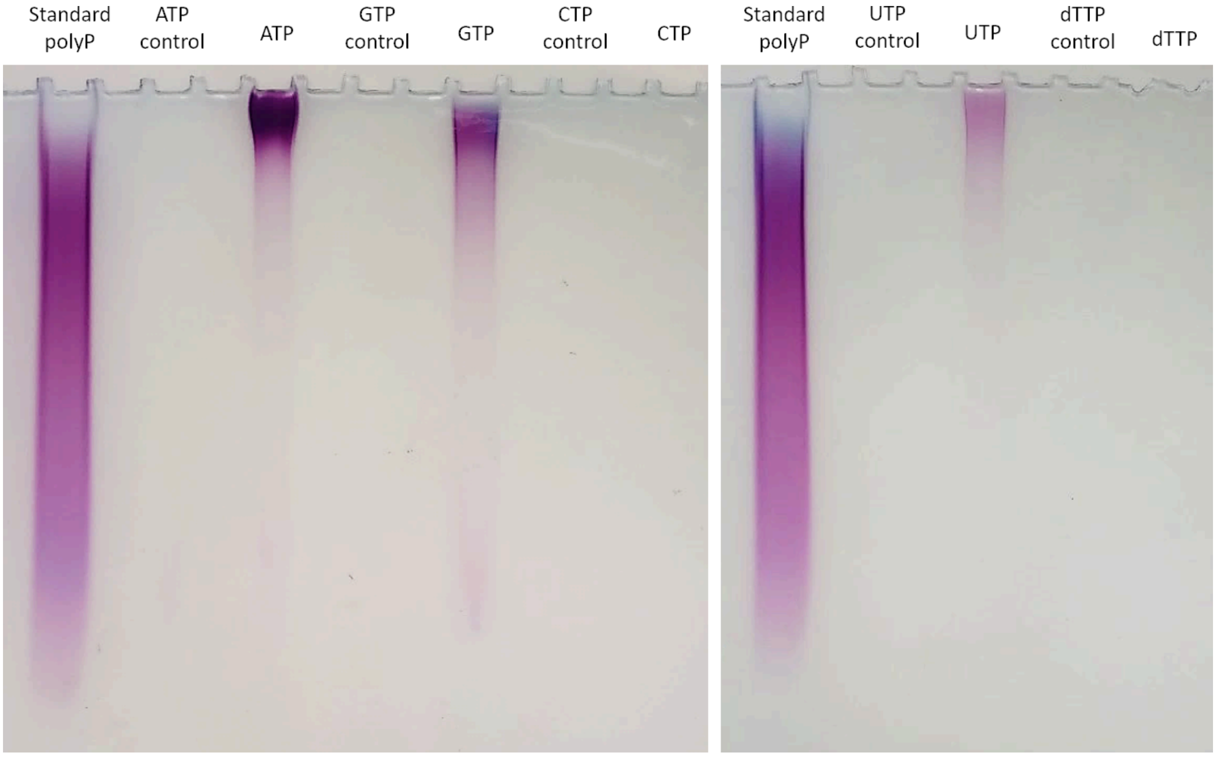

Online Resource 5a: Separation of $PPK2_{AT}$-produced polyP after **30 min reaction time** by PAGE and subsequent toluidine staining. Standard polyP (average chain length≈100 P_i residues corresponding to 9 mM P_i) (lane 1 and 8), 15 mM of ATP, GTP, CTP, UTP or dTTP (lanes 3, 5, 7, 10 and 12), controls with 15 mM ATP, GTP, CTP, UTP or dTTP but without $PPK2_{AT}$ (lane 2, 4, 6, 9, 11).

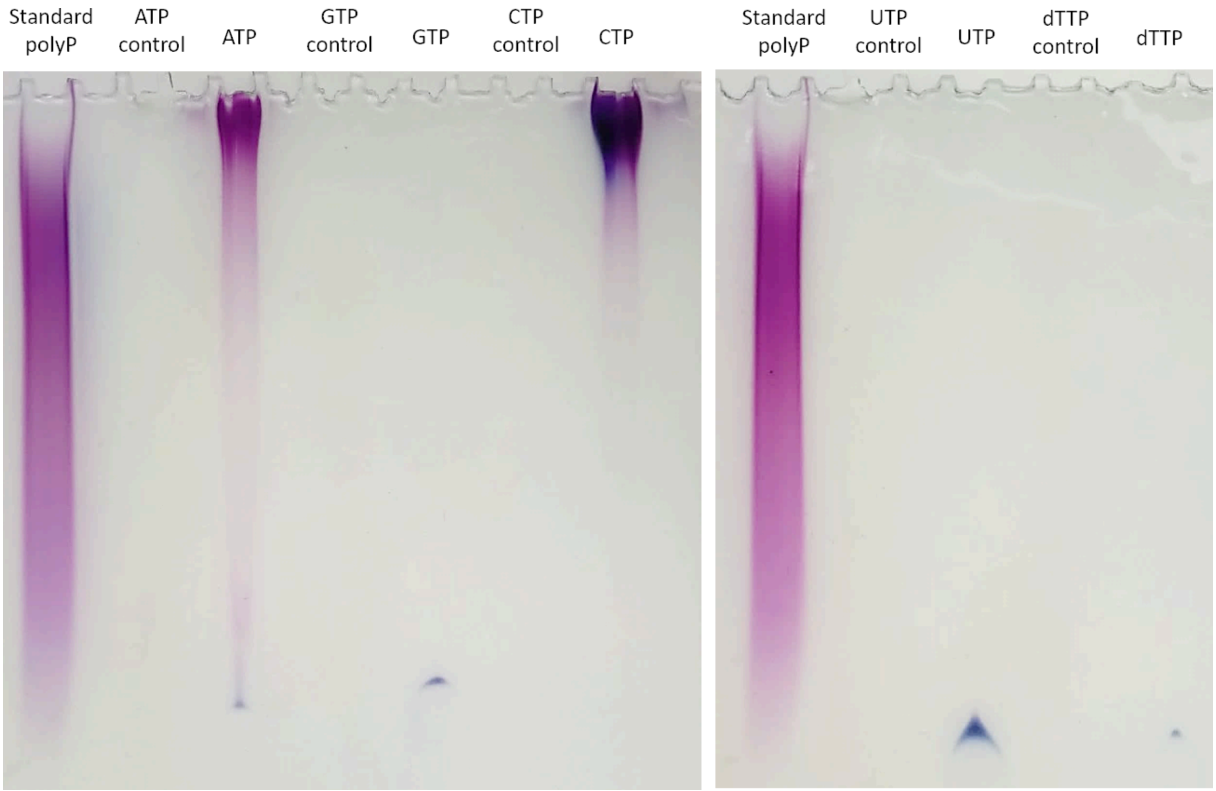

Online Resource 5b: Separation of $PPK2_{AT}$ produced polyP **after 24h reaction time** by PAGE and subsequent toluidine staining. Standard polyP (average chain length≈100 P_i residues corresponding to 9 mM P_i) 15 mM of ATP, GTP, CTP, UTP or dTTP were used as substrate as indicated. The control lanes were performed without enzyme.

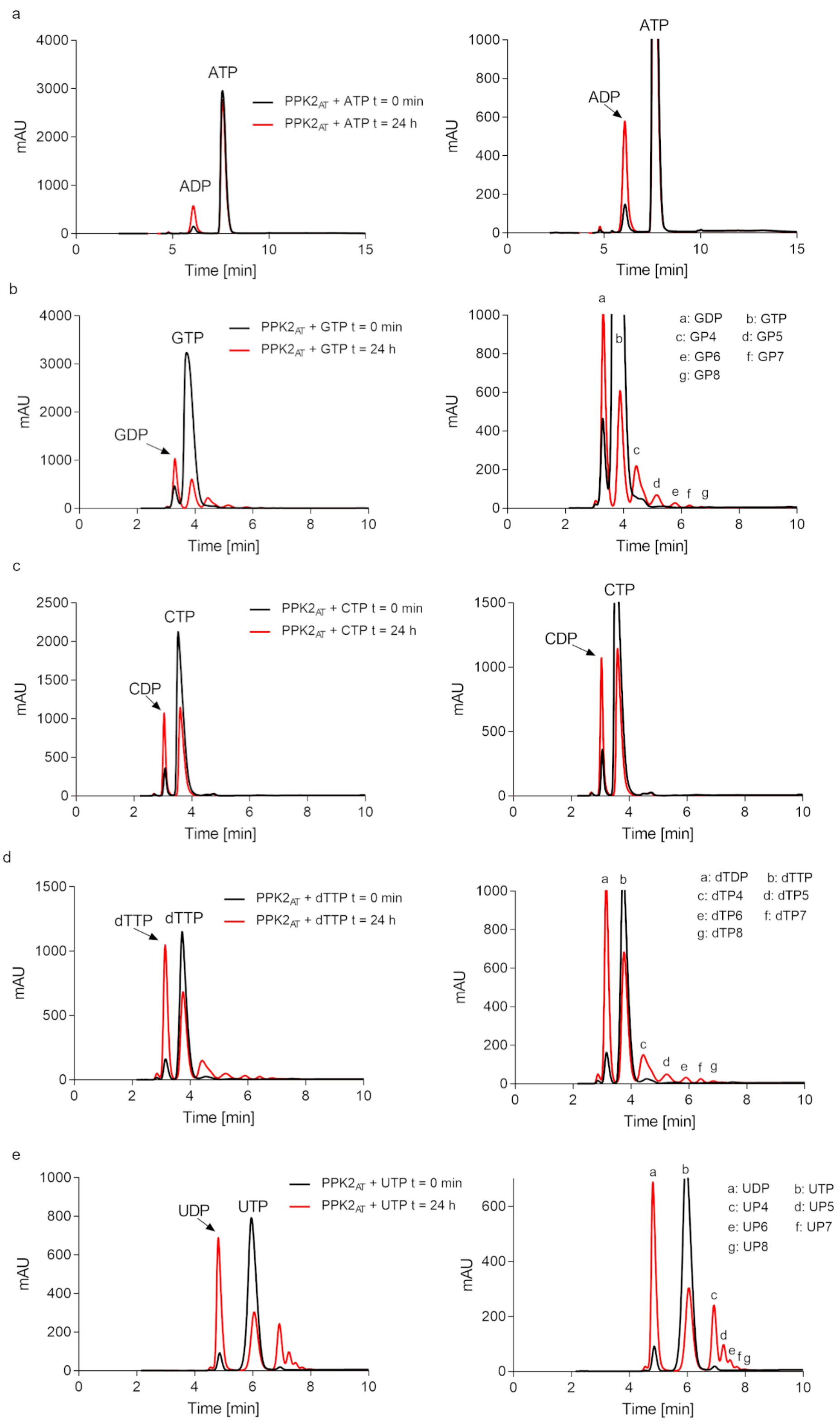

Online resource 6: Formation of oligo-phosphorylated nucleosides by $PPK2_{AT}$. The reaction products of $PPK2_{AT}$ after 24 h incubation NTPs as indicated were analyzed by HPLC. The left images show the concentrations of the educt and product nucleotides before (0 min, black) and after the addition of $PPK2_{AT}$ (24h, red, mAU, milli-absorption units at 254 nm). The images on the right represent enlargements of the same chromatograms to better visualize the small peaks of produced oligophosphorylated nucleosides.

Online resource 7: Overview of $PPK2_{AT}$ enzyme assay results.

Nucleotide (15mM)	Cofactor (2mM)	Incubation time	Product	PolyP gel
ATP	$MnCl_2$	30 min	ADP (++)	+
GTP	$MnCl_2$	30 min	GDP (++)	+
CTP	$MnCl_2$	30 min	CDP (-)	-
dTTP	$MnCl_2$	30 min	dTDP (-)	-
UTP	$MnCl_2$	30 min	UDP (+)	+
ATP	$MgCl_2$	30 min	ADP (+)	-
GTP	$MgCl_2$	30 min	GDP (+)	-
CTP	$MgCl_2$	30 min	CDP (+)	-
dTTP	$MgCl_2$	30 min	dTDP (+)	-
UTP	$MgCl_2$	30 min	UDP (+)	-
ATP	$MnCl_2$	24h	ADP (+), AP4	+
GTP	$MnCl_2$	24h	GDP (++) , GP4, GP6, GP7	-
CTP	$MnCl_2$	24h	CDP (++), CP4	+
dTTP	$MnCl_2$	24h	dTDP (++) , dTP4, dTP6, dTP7, dTP8	-
UTP	$MnCl_2$	24h	UDP (++), UP4, UP6, UP7, UP8, UP9	-
ATP	$MgCl_2$	24h	ADP (+)	-
GTP	$MgCl_2$	24h	GDP (+)	-
CTP	$MgCl_2$	24h	CDP (+)	-
dTTP	$MgCl_2$	24h	dTDP (+)	-
UTP	$MgCl_2$	24h	UDP (+)	-
ADP + polyP	$MnCl_2$	30 min	ATP (+), AP4	N.D.
GDP + polyP	$MnCl_2$	30 min	GTP (++), GP4	N.D.
CDP + polyP	$MnCl_2$	30 min	CTP (++)	N.D.
dTDP + polyP	$MnCl_2$	30 min	dTTP (++	N.D.
UDP + polyP	$MnCl_2$	30 min	UTP (++), UP4, UP6, UP7, UP8	N.D.
ADP + polyP	$MgCl_2$	30 min	ATP (-)	N.D.
GDP + polyP	$MgCl_2$	30 min	GTP (++), GP4	N.D.
CDP + polyP	$MgCl_2$	30 min	CTP (+), CP4	N.D.
dTDP + polyP	$MgCl_2$	30 min	dTTP (+), dTP4	N.D.
UDP + polyP	$MgCl_2$	30 min	UTP (+)	N.D.
ADP + polyP	$MnCl_2$	24h	ATP (+), AP4	N.D.
GDP + polyP	$MnCl_2$	24h	GTP (++), GP4, GP6, GP7, GP8	N.D.
CDP + polyP	$MnCl_2$	24h	CTP (++), CP4, CP6, CP7, CP8	N.D.
dTDP + polyP	$MnCl_2$	24h	dTTP (++), dTP4, dTP6, dTP7, dTP8	N.D.
UDP + polyP	$MnCl_2$	24h	UTP (++), UP4, UP6, UP7, UP8	N.D.
ADP + polyP	$MgCl_2$	24h	ATP (-)	N.D.
GDP + polyP	$MgCl_2$	24h	GTP (++), GP4	N.D.
CDP + polyP	$MgCl_2$	24h	CTP (+), CP4	N.D.
dTDP + polyP	$MgCl_2$	24h	dTTP (+), dTP4	N.D.
UDP + polyP	$MgCl_2$	24h	UTP (+)	N.D.

(+), (++) and (-) in the product column means that the respective nucleotide peak area was low, high or absent, respectively. (+) and (-) in the PolyP gel column indicates the presence or absence of a polyP-toluidine signal after electrophoresis; (N.D., not determined).

Online resource 8: HILIC-QQQ-SIM Total Ion Chromatograms (TIC) and Extracted Ion Chromatograms (EIC) of selected NTP Assays and related 100 µM Standard Mixes (see also Table 2)

(a) APx 100 µM Std-Mix (TIC)
TIC of the adenosine nucleotides (AMP, ADP, ATP) standard mixture. Peak heights [%] were normalized to the most abundant peak.

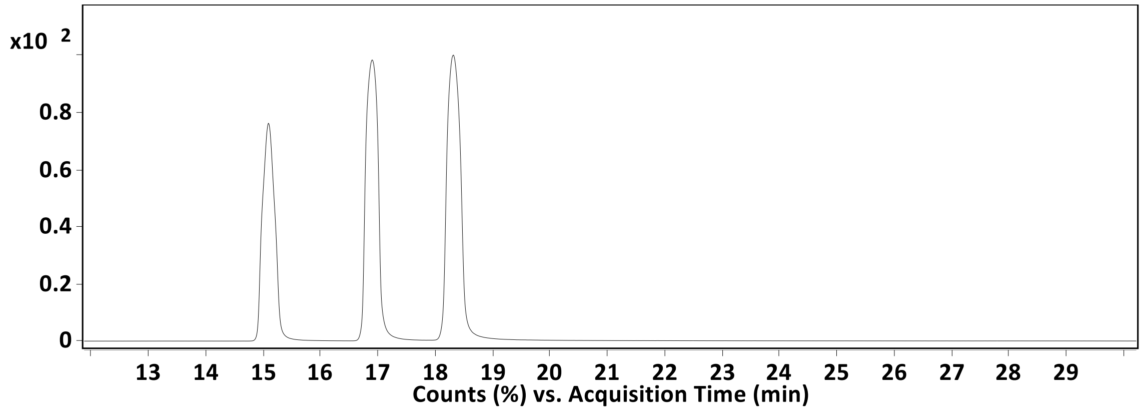

(b) APx 100 µM Std-Mix (EIC)
EIC of targeted SIM transitions. Peaks are labeled with areas [Cts] and rentention times [min].

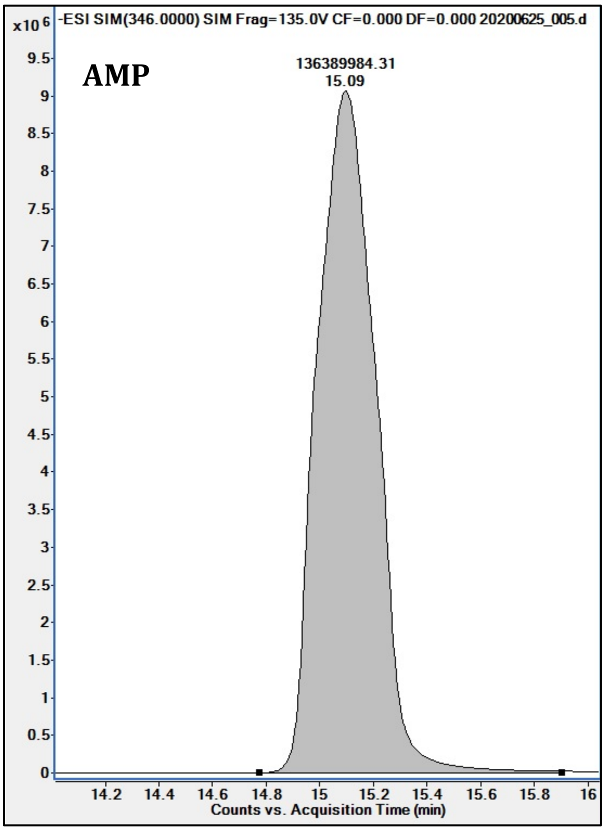

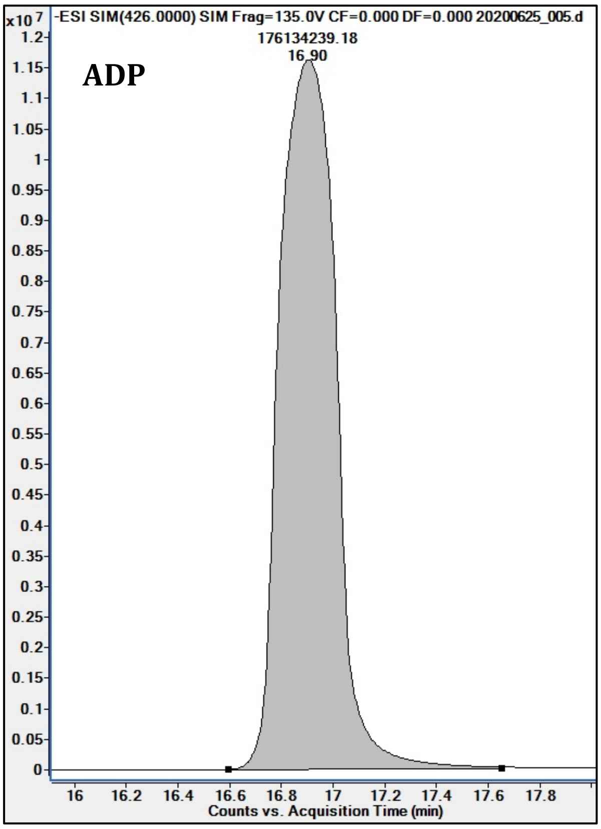

(b) APx 100 µM Std-Mix (EIC) (continued)

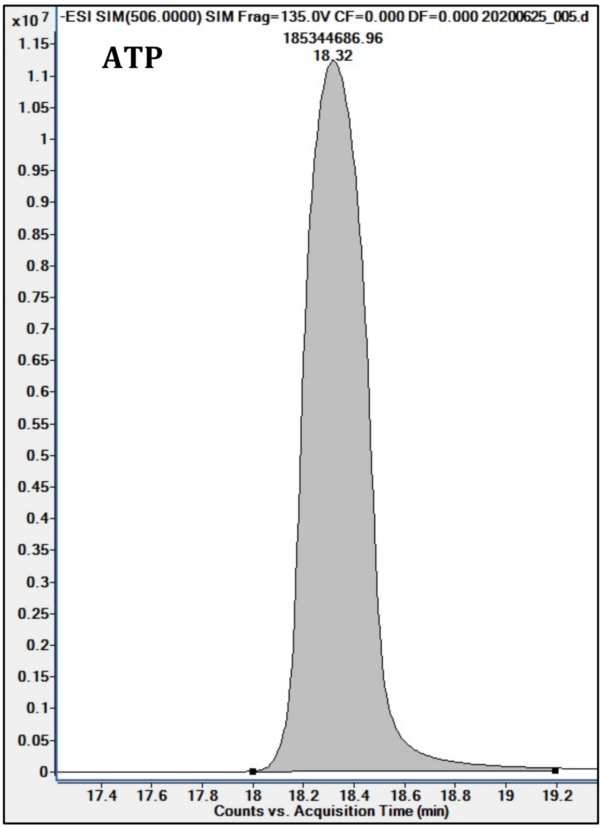

(c) APx PPK2$_{AT}$ Assay (TIC)

TIC of the PPK2$_{AT}$ ATP assay. Peak heights [%] were normalized to the most abundant peak.

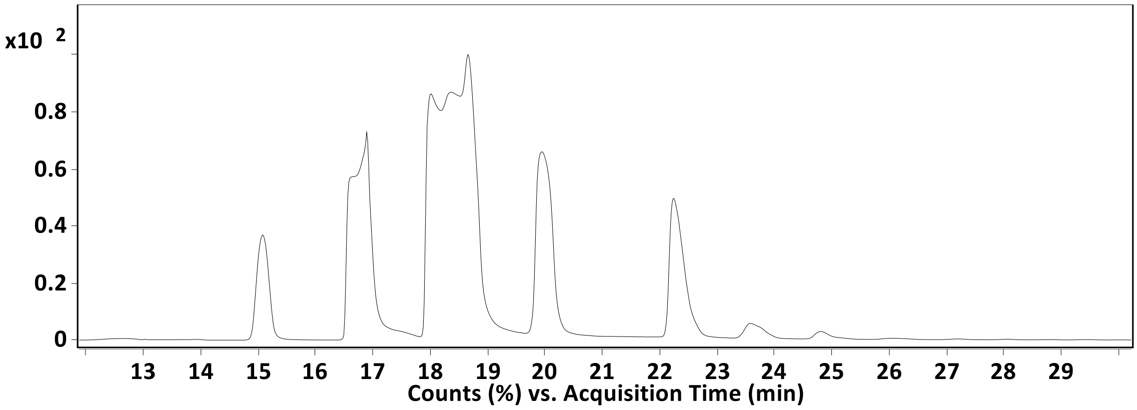

(d) APx PPK2$_{AT}$ Assay (EIC)

EIC of targeted SIM transitions. Peaks are labeled with areas [Cts] and rentention times [min].

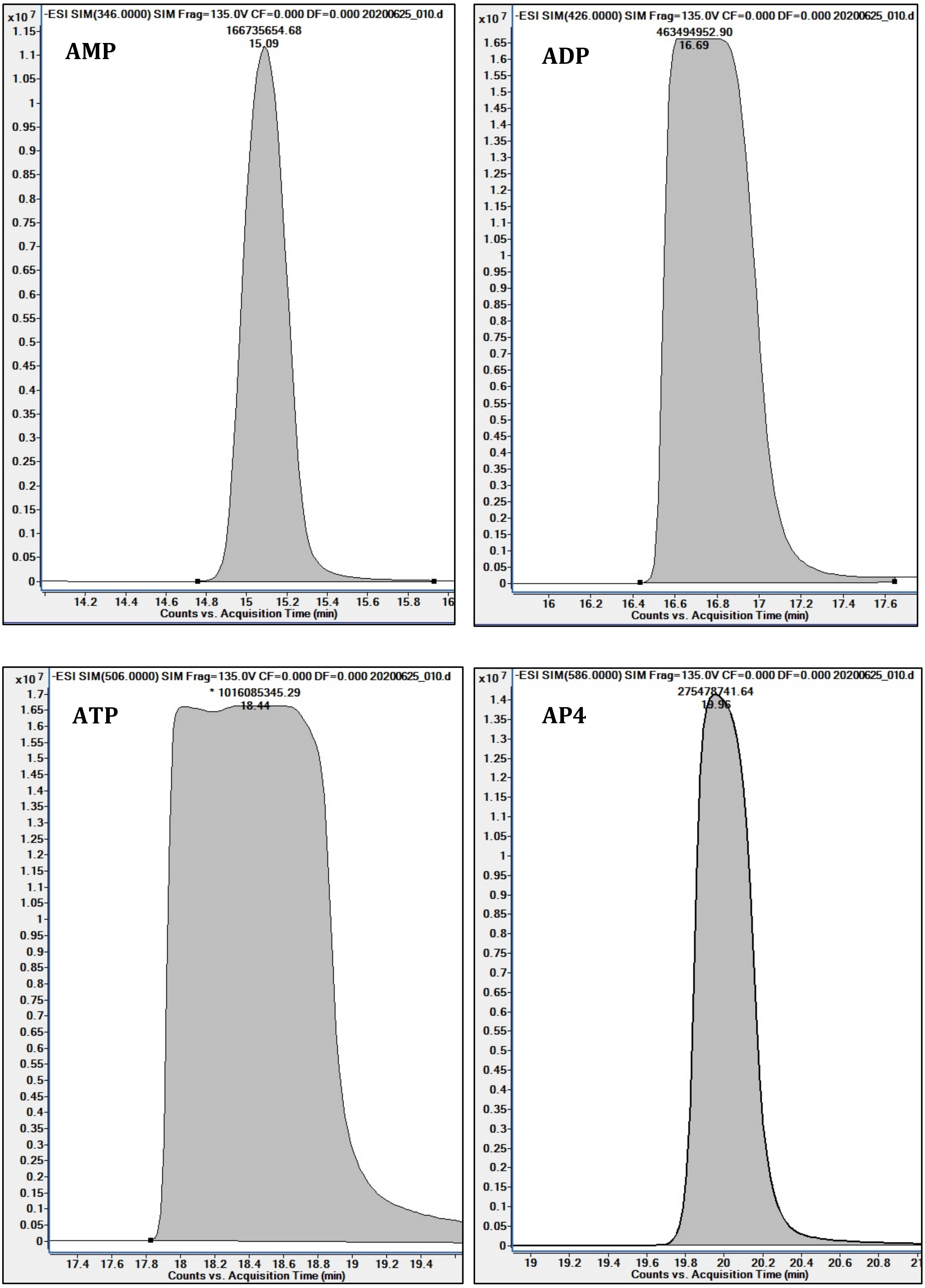

(d) APx PPK2$_{AT}$ Assay (EIC) (continued)

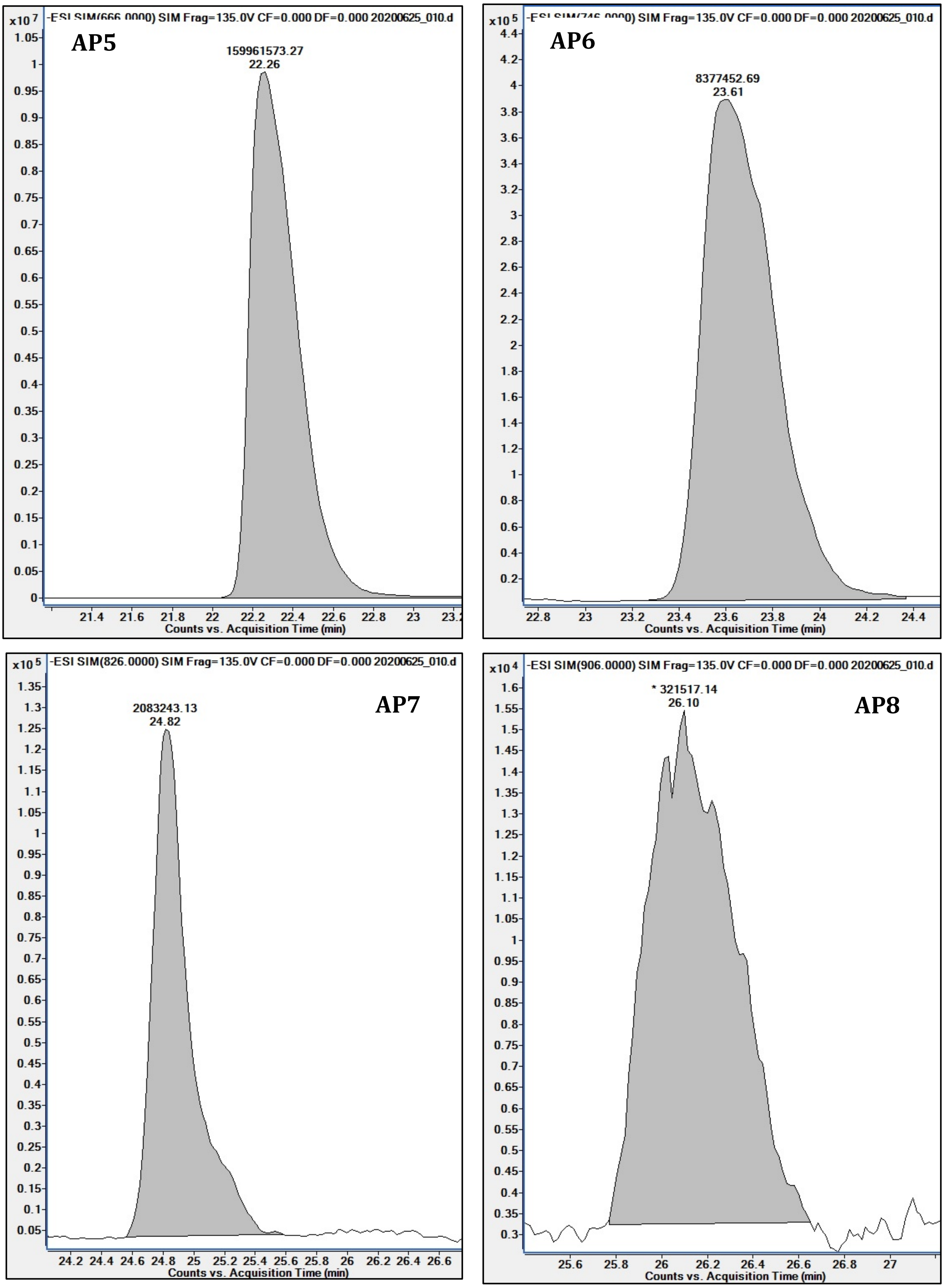

(d) APx PPK2$_{AT}$ Assay (EIC) (continued)

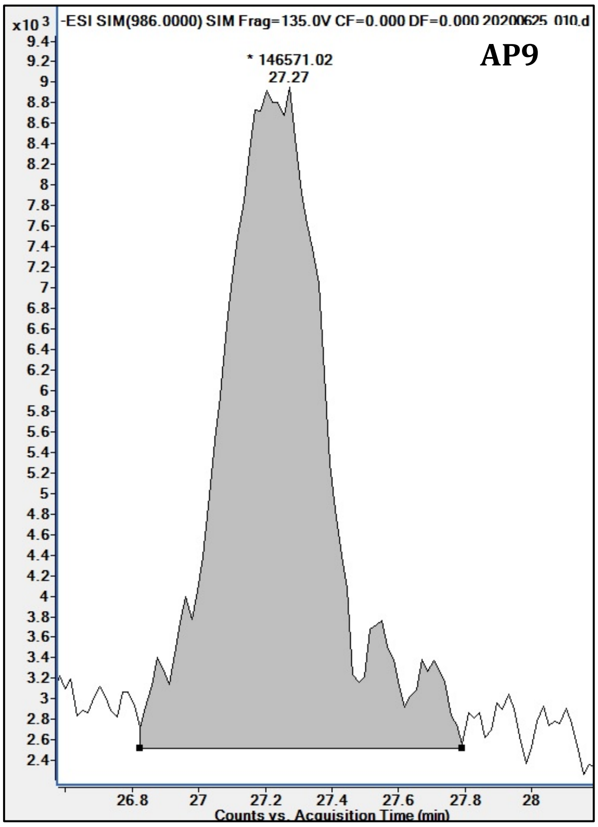

(e) GPx 100 µM Std-Mix (TIC)

TIC of the guanosine nucleotides standard mixture (GMP, GDP, GTP). Peak heights [%] were normalized to the most abundant peak.

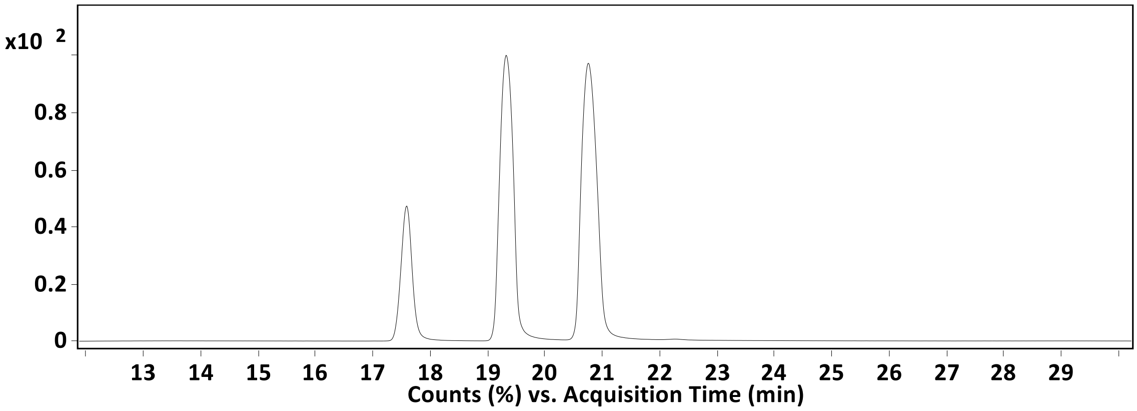

(f) GPx 100 µM Std-Mix (EIC)

EIC of targeted SIM transitions. Peaks are labeled with areas [Cts] and rentention times [min].

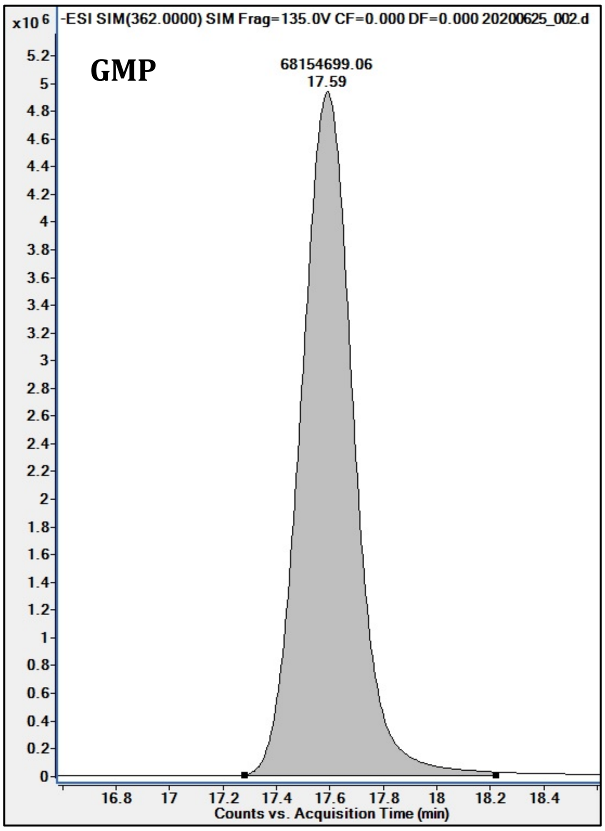

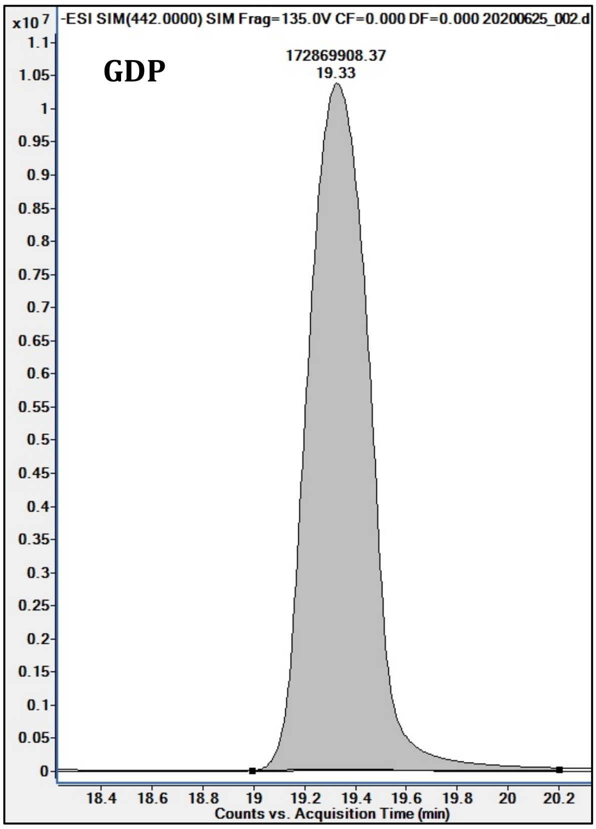

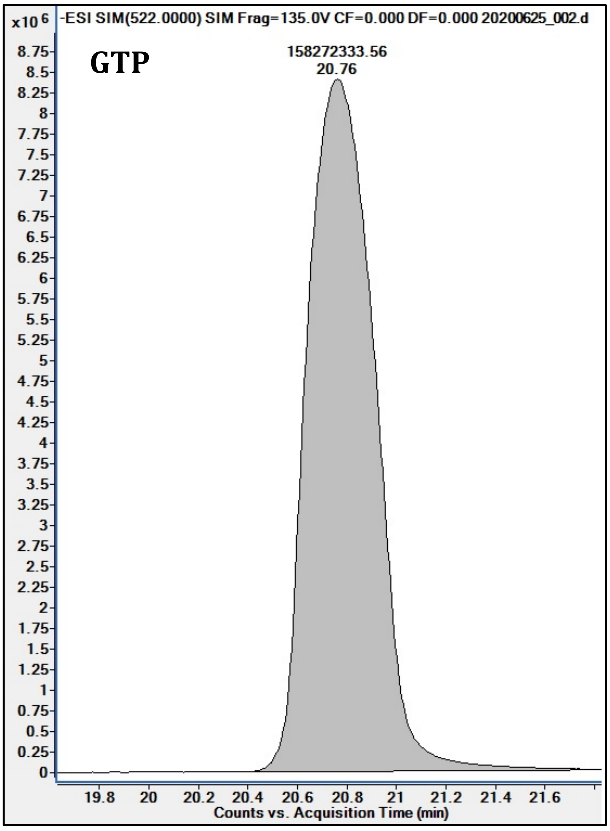

(g) GPx PPK2$_{AT}$ Assay (TIC)

TIC of the PPK2$_{AT}$ GTP assay. Peak heights [%] were normalized to the most abundant peak.

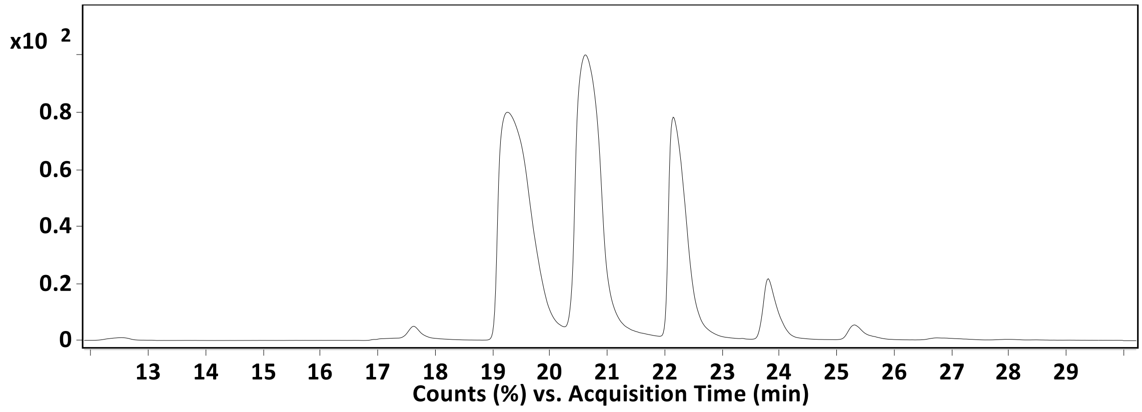

(h) GPx PPK2$_{AT}$ Assay (EIC)

EIC of targeted SIM transitions. Peaks are labeled with areas [Cts] and rentention times [min].

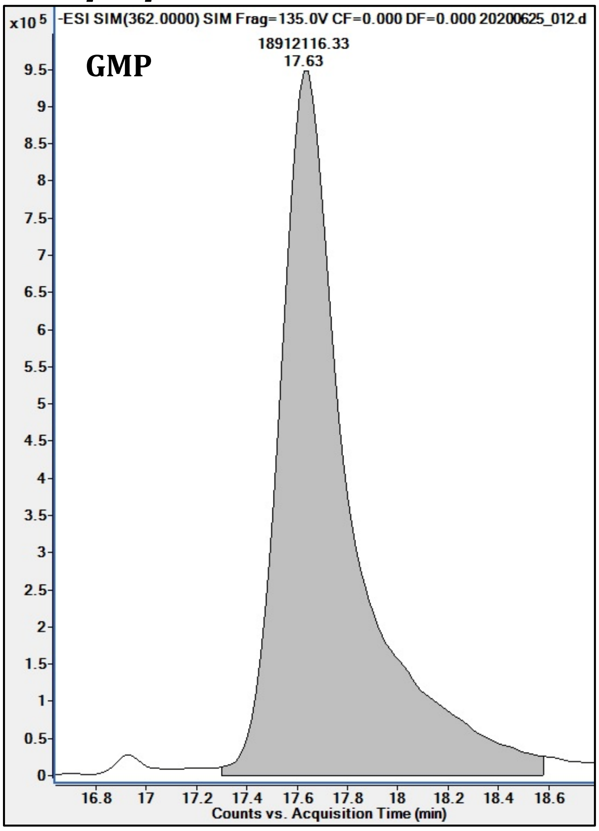

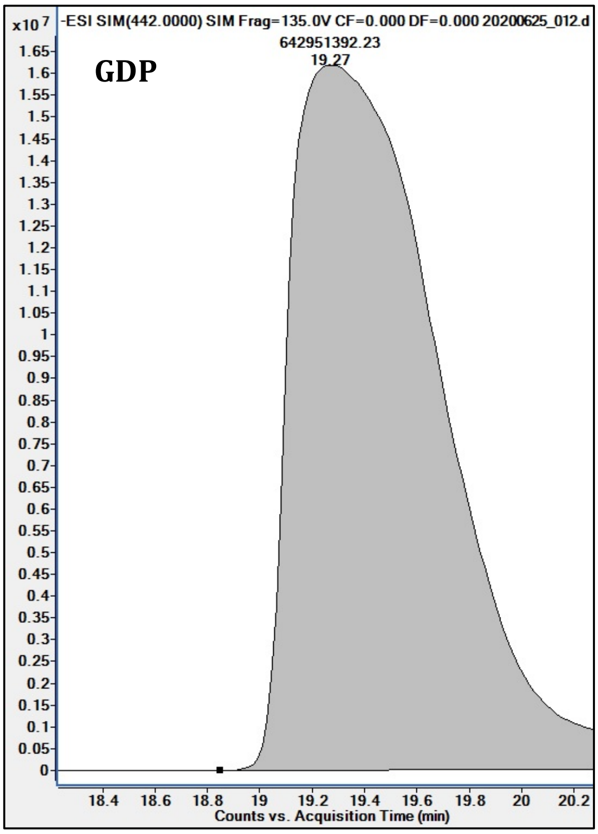

(h) GPx PPK2$_{AT}$ Assay (EIC) (continued)

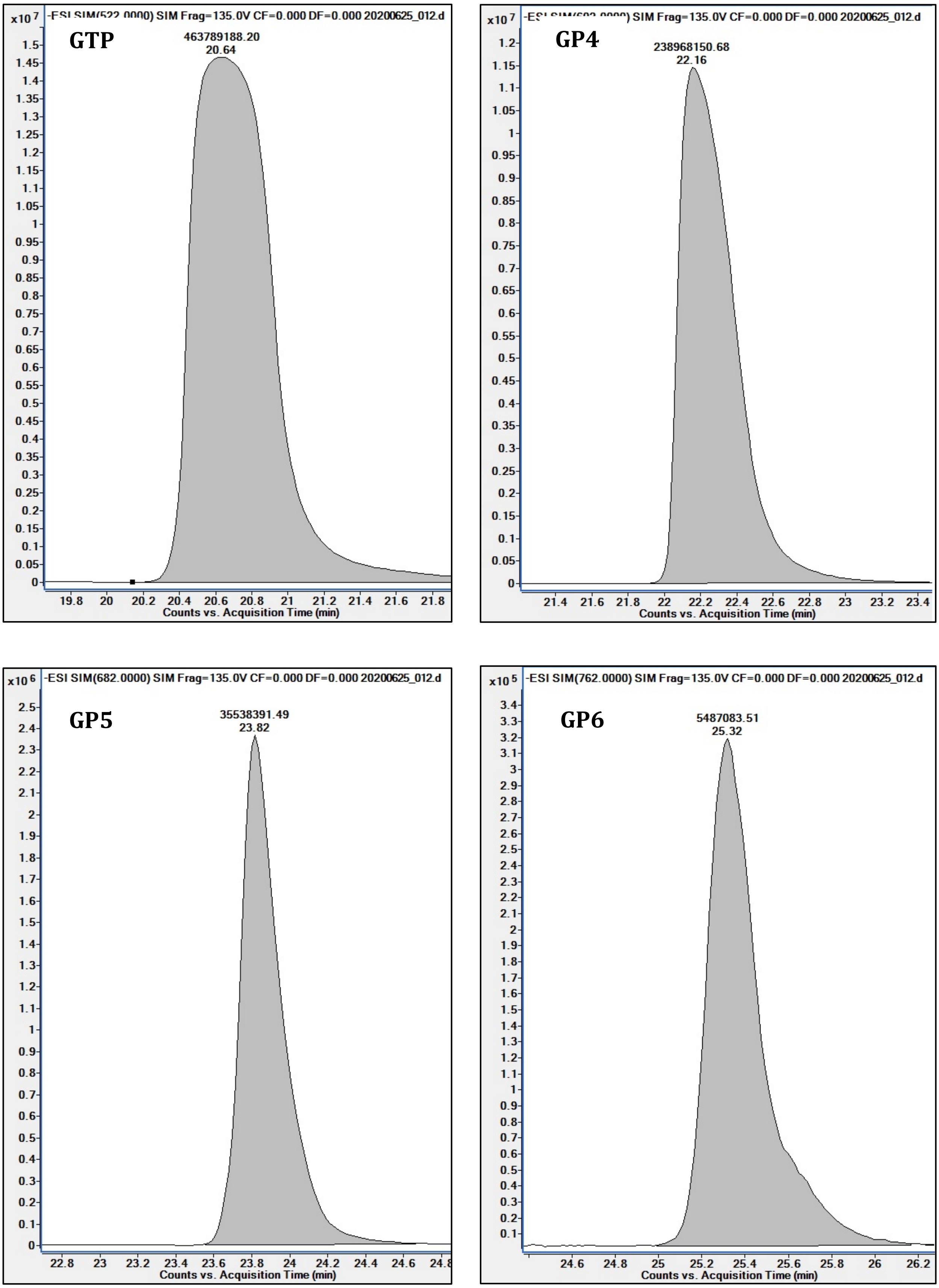

(h) GPx $PPK2_{AT}$ Assay (EIC) (continued)

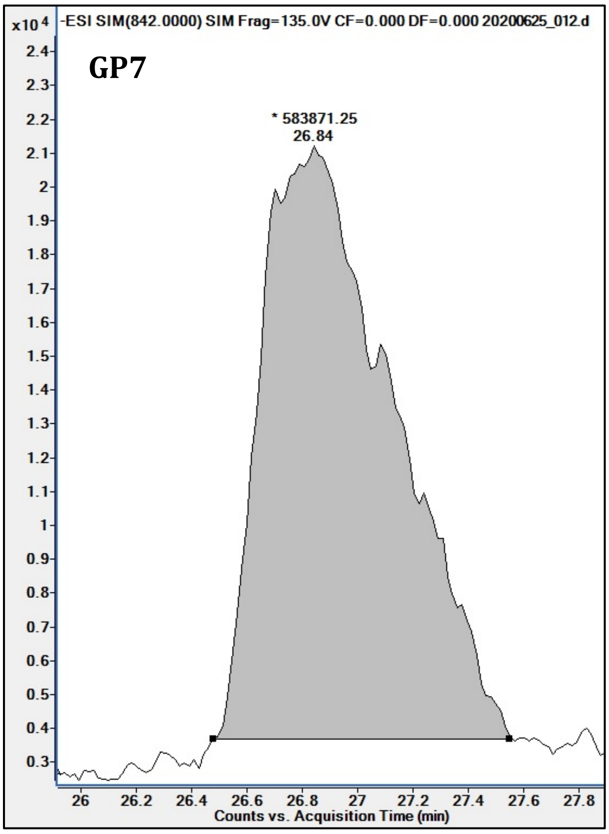

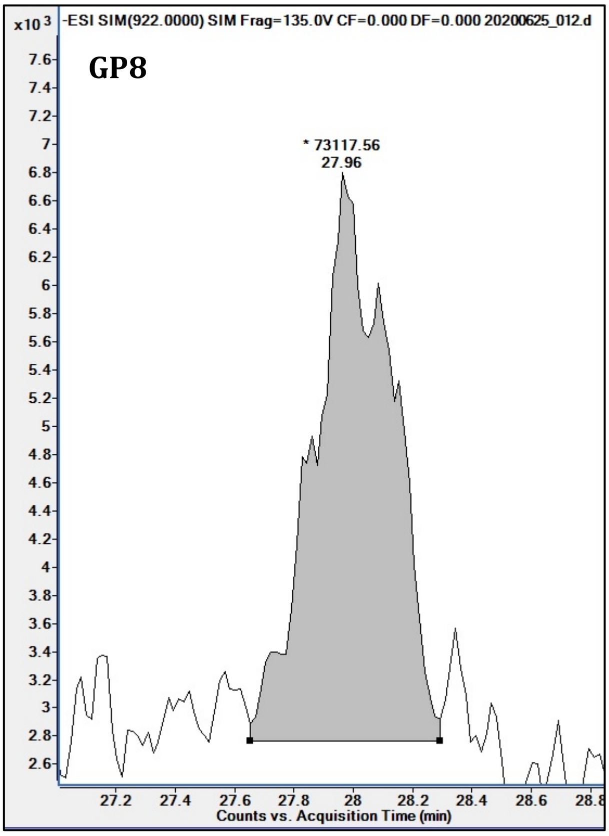

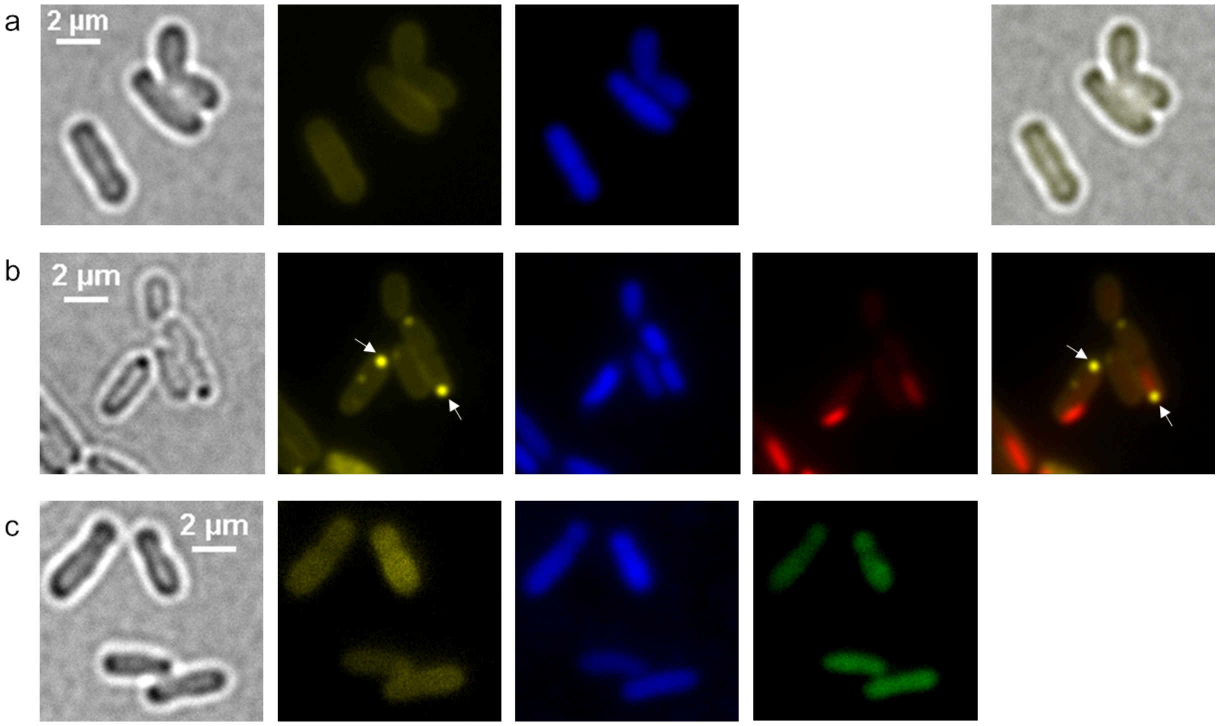

Online resource 9: Formation of polyP by PPK1$_{AT}$-mCherry in *A. tumefaciens Δppk1 Δppk2*. LB-grown cells were stained with DAPI and imaged in bright field (left), fluorescence microscopically with a (second left) DAPI-polyP-specific or DAPI-DNA-specific (third right) filter. (**a**) Deletion of *ppk1* and *ppk2* results in a phenotype with no DAPI-stainable polyP-granules. However, *A. tumefaciens Δppk1 Δppk2* cells harbouring pBBR1MCS2::P*phaC-ppk1$_{AT}$-mCherry*, shown in (**b**) are able to form DAPI-stainable polyP-granules again (indicated by arrows) that can be located either close to the PPK1-mCherry signal or not. From left to right: bright field, DAPI-polyP channel, DAPI-DNA channel and mCherry channel. (**c**) Introduction of pBBR1MCS2::P*phaC-eyfp-ppk2$_{AT}$* into the *ppk1 ppk2* deletion strain of *A. tumefaciens* caused a homogenous fluorescence of eYFP-PPK2 and no formation of DAPI stainable polyP. From left to right: bright field, DAPI-polyP channel, DAPI-DNA channel and eYFP channel.
